# *COPS5* amplification and overexpression confers tamoxifen-resistance in ERα-positive breast cancer by degradation of NCoR

**DOI:** 10.1038/ncomms12044

**Published:** 2016-07-04

**Authors:** Renquan Lu, Xiaobo Hu, Junmei Zhou, Jiajun Sun, Alan Z. Zhu, Xiaofeng Xu, Hui Zheng, Xiang Gao, Xian Wang, Hongchuan Jin, Ping Zhu, Lin Guo

**Affiliations:** 1 Department of Clinical Laboratory, Shanghai Cancer Center, Fudan University; Department of Oncology, Shanghai Medical School, Fudan University, Shanghai 200032, China; 2 Department of Laboratory Medicine, Long Hua Hospital, Shanghai University of TCM, Shanghai 200032, China; 3 Gerchi Biotech Research Laboratories, 100 Research Park, Zhejiang 314300, China; 4 Department of Medical Oncology, Sir Run Run Shaw Hospital, Zhejiang 310016, China

## Abstract

Oestrogen receptor α (ERα) antagonists are used in endocrine therapies for ERα-positive (ERα+) breast cancer patients. Unfortunately the clinical benefit is limited due to intrinsic and acquired drug resistance. Here using integrated genomic and functional studies, we report that amplification and/or overexpression of *COPS5* (CSN5/JAB1) confers resistance to tamoxifen. Amplification and overexpression of COPS5, a catalytic subunit of the COP9 complex, is present in about 9% of the ERα+ primary breast cancer and more frequently (86.7%, 26/30) in tamoxifen-refractory tumours. Overexpression of COPS5, through its isopeptidase activity, leads to ubiquitination and proteasome-mediated degradation of NCoR, a key corepressor for ERα and tamoxifen-mediated suppression of ERα target genes. Importantly, COPS5 overexpression causes tamoxifen-resistance in preclinical breast cancer models *in vitro* and *in vivo*. We also demonstrate that genetic inhibition of the isopeptidase activity of COPS5 is sufficient to re-sensitize the resistant breast cancer cells to tamoxifen-treatment, offering a potential therapeutic approach for endocrine-resistant breast cancer patients.

## Abstract (web summary)

The corepressor NCoR is required for tamoxifen-mediated ERα-dependent transcriptional repression. Here, the authors show that COPS5 confers tamoxifen-resistance through the degradation of NCOR, the recruitment of the co-activator PCAF to ERα binding sites and the subsequent ERα transcriptional activity.

## Graphical abstract

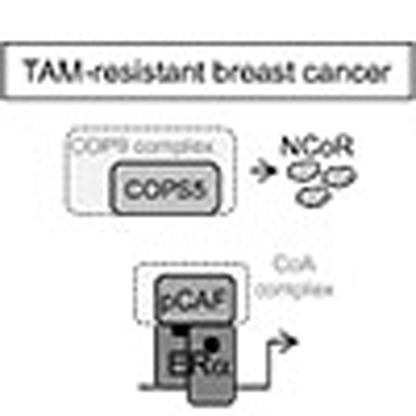

Breast cancer is the most common cancer in women worldwide, with nearly 1.7 million new cases diagnosed each year. WHO (World Health Organization) estimated that worldwide over 521,000 women died in 2012 due to breast cancer. About 80% of the breast cancer patients express oestrogen receptor α (ERα) in their tumours, which is essential for normal mammalian gland development and breast cancer development and progression. Therefore, ERα antagonists, for example, tamoxifen and fulvestrant, and aromatase inhibitors (AIs) that block the ER signalling are widely used in endocrine therapies for ERα-positive (ERα+) breast cancer patients^1^,^2^,^3^. Tamoxifen is the most prescribed ER antagonist particularly in the first-line therapy. Unfortunately the clinical benefit is limited because of intrinsic and acquired drug resistance. The appearance and development of resistant tumours are major obstacles in controlling the growth and metastasis of breast cancer. A number of mechanisms have been associated with resistance to tamoxifen^4^,^5^. Activation of the receptor tyrosine kinases, particularly ERBB2/HER2, EGFR and IGF1R, and other kinase pathways, for example, PI3K/AKT/mTOR, have been shown to contribute to endocrine-resistance^6^,^7^,^8^. Apoptosis and cell cycle regulators appear to play roles in refractory to endocrine therapy in breast cancer as well^8^,^9^. Dysregulation of ERα coregulators, including coactivators p160s/NCoAs and corepressors NCoR and SMRT, have been shown to be crucial in switching antagonistic selective ER modulators (SERMs) to agonists^10^,^11^. In addition, hotspot mutations in the ligand-binding domain of ERα, which confer partial resistance to current endocrine therapies including AIs, SERMs and selective ER degraders (SERDs), were observed in ∼20% of the endocrine-refractory metastatic breast cancer^12^,^13^,^14^,^15^,^16^. Activation of the kinase and anti-apoptotic pathways, loss of ERα expression by genetic or epigenetic mechanisms, stem cells or epithelial–mesenchymal transition (EMT), and autophagocytosis can also account for ERα-independent resistance to endocrine therapies^4^,^17^. Together, it appears that breast cancer can utilize multiple molecular approaches to development of endocrine therapy refractory. Uncovering novel mechanisms that are prevalent in resistant tumours is of importance to develop new therapeutic strategies for drug-resistant patients.

Here using integrated genomic and functional studies, we report that amplification and/or overexpression of the COP9 complex-associated isopeptidase COPS5 is presented in about 9% of the ERα+ primary breast cancer and more frequently (86.7%, 26/30) in tamoxifen-resistant tumours. Mechanistically, amplification and overexpression of COPS5 lead to an ubiquitination/proteasome-dependent destabilization of NCoR protein, a corepressor required for tamoxifen-mediated suppression of ERα target genes, which is considered as a key mechanism of action of the drug. In turn, COPS5 overexpression confers resistance to tamoxifen by converting it to a potent ERα agonist. We also show evidence that inhibition of the isopeptidase activity of COPS5 is sufficient to re-sensitize the resistant breast cancer cells to tamoxifen-treatment, providing a potential therapeutic approach for endocrine-resistant breast cancer patients.

## Results

### COPS5 overexpression in tamoxifen-resistant breast cancer

**Figure 1:**
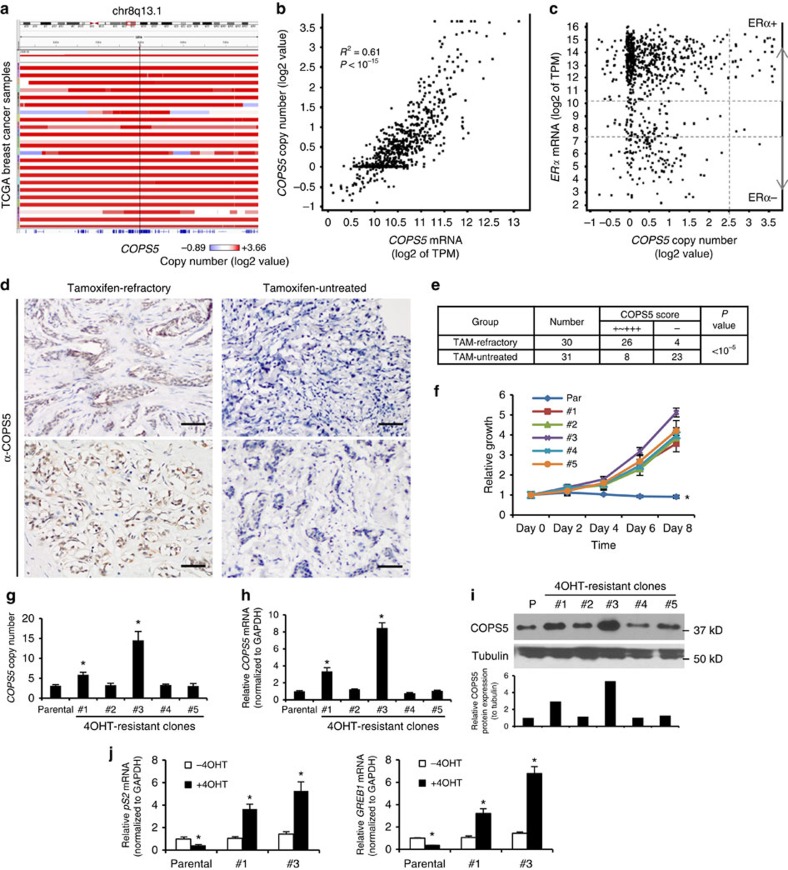
*COPS5* is amplified and overexpressed in ERα+ breast cancer and tamoxifen-resistant MCF7 cells. (**a**) Focal amplification of *COPS5* in TCGA breast cancer. Samples were sorted by *COPS5* amplitude from the IGV of TCGA portal developed by the Broad Institute. (**b**) Correlation of mRNA level and copy number of *COPS5* in TCGA breast cancer samples. (**c**) *COPS5* copy number and ERα mRNA expression in TCGA breast cancer samples. (**d**) Immunohistochemistry of COPS5 in representative tamoxifen-untreated and refractory breast cancer. Anti-COPS5 antibody showed positive staining in the nucleus of tumour cells (scale bar, 50 μm). (**e**) *χ*^2^-calculation of the significance of COPS5-overexpression rates in tamoxifen-refractory and tamoxifen-untreated breast tumours. (**f**) Growth curve of parental and tamoxifen-resistant MCF7 clones established by *in vitro* dose-escalation 4OHT-treatment (refer to Methods) in the presence of 2 μM of 4OHT. Values are normalized to day 0 treatment. *Significant difference compared with other samples (*P*<0.01 at both day 6 and day 8, Student's *t*-test). (**g**) Copy number of *COPS5* genomic DNA normalized to *LINE1* in parental and tamoxifen-resistant MCF7 clones. *Significant difference compared with parental MCF7 (*P*<0.01, Student's *t*-test). (**h**) Expression of *COPS5* mRNA in parental and tamoxifen-resistant MCF7 clones. Data were normalized to *GAPDH* and relative to *COPS5* expression in the parental cell line which was set as 1. *Significant difference compared with parental MCF7 (*P*<0.01, Student's *t*-test). (**i**) Western blot analysis and quantification of COPS5 protein expression in parental and tamoxifen-resistant MCF7 clones. (**j**) Quantitative RT–PCR analysis of ERα target genes *pS2* and *GREB1* in MCF7 clones after treatment with 500 nM 4OHT for 48 h. Data are presented as mean+s.e.m. of three biological replicates. *Significant difference compared with untreated parental MCF7 (*P*<0.01, Student's *t*-test).

To explore the clinically relevant mechanisms associated with tamoxifen-resistance, we analysed both The Cancer Genome Atlas (TCGA) breast cancer data, which contains mainly primary and untreated or lightly treated patient samples, and our internal breast patient samples collected pre- and post-tamoxifen-treatment. *COPS5* is one of the frequently altered genes in ERα+ breast cancer samples from TCGA. *COPS5* gene is located at the chromosome 8q13 which is amplified (copy number (CN)>6) in about 9% (70/774) of the ERα+ breast cancer (Log2 *ESR1* expression>5). Although in general the amplicon is relatively broad and not scored as a significant GISTIC peak in a recent pan- or lineage-specific TCGA cancer analysis^18^,^19^, there is a trend of *COPS5*-centric focality, particularly in the patients with high copy number of *COPS5* and focal amplification (Fig. 1a). Importantly, the expression of *COPS5* mRNA correlates with the CN (*R*^2^=0.61, Pearson's *χ*^2^-test *P*<10^−15^), suggesting a potential functional relevance of *COPS5* amplification (Fig. 1b). Of note, high levels of amplitude (CN>12) of *COPS5* are significantly enriched in the ERα+ breast cancer patients, implicating that the amplification and overexpression of *COPS5* may contribute to the ERα-related signalling and tumour progression (Fig. 1c, Supplementary Table 1). In addition, the clinical outcomes (disease-free survival rate and overall survival rate) of the TCGA breast cancer patients (including luminal A and B) with high expression levels of *COPS5* appeared to be poorer (Supplementary Fig. 1a,b). In addition, the worse clinical outcomes are unlikely to be associated with tumour subtypes because *COPS5* expression was similar in luminal A and B tumours (Supplementary Fig. 1c). To evaluate if COPS5 protein overexpression is involved in tamoxifen-resistance, we investigated ERα+ breast tumours that were untreated (*n*=31) or refractory to tamoxifen-treatment (*n*=30). Notably, COPS5 is mainly expressed in cellular nucleus of cancer cells (Fig. 1d). Based on the immunohistochemistry (IHC) staining scores (refer to Methods), the rate of high expression (+ to +++) of COPS5 protein is significantly elevated in tamoxifen-refractory breast tumours (86.7%, 26/30) compared with untreated breast tumours (25.8%, 8/31) (Fig. 1d,e, Supplementary Table 2). To further evaluate the role of COPS5 in a model system, we attempted to establish *in vitro* models derived from MCF7 ERα+ breast cancer cells that are highly resistant to 4-hydroxy-tamoxifen (4OHT, an active metabolite of tamoxifen) at up to 2 μM by a dose-escalation protocol (refer to Methods) (Fig. 1f). These clones are also partially resistant to the SERD fulvestrant but not the pan-cytotoxic CDK inhibitor flavopiridol (Supplementary Fig. 2), suggesting that resistance mechanism is specific to antiestrogens. Strikingly, among five resistant clones, we identified two clones with amplified CN of *COPS5* (Fig. 1g), and increased mRNA (Fig. 1h) and protein expression levels of COPS5 (Fig. 1i). Furthermore, 4OHT showed potent agonistic instead of antagonistic or weak agonistic activity in both clones as tested by the expression of six ERα target genes (Fig. 1j, Supplementary Fig. 3), suggesting a general role of COPS5 overexpression in activation of ERα-dependent transcription in response to 4OHT. Therefore, the clones #1 and #3 recapitulated COPS5 overexpression observed in clinical tamoxifen-refractory tumours, and were used for further studies of the mechanisms of tamoxifen-resistance. In summary, in both clinical breast tumour samples and *in vitro* cell line models we observed that amplification and overexpression of *COPS5* was associated with endocrine/tamoxifen-resistance.

### Overexpression of COPS5 confers resistance to tamoxifen

**Figure 2:**
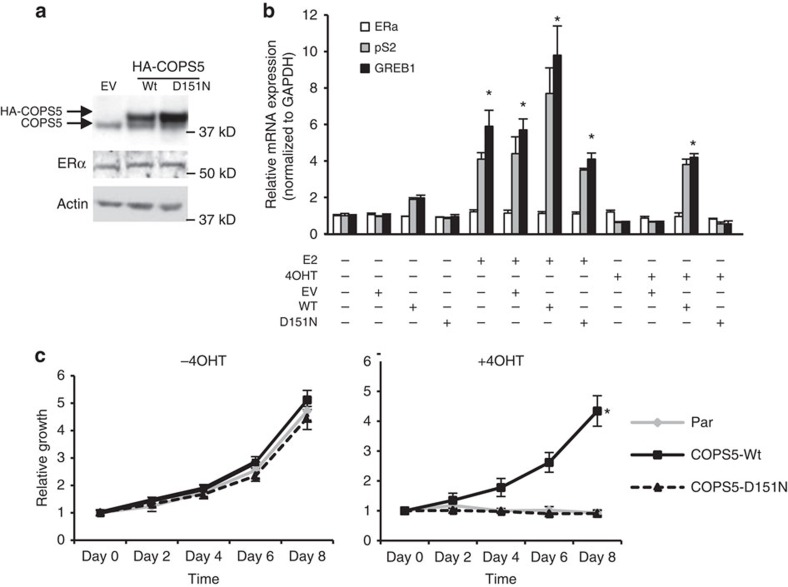
Overexpression of wild-type COPS5 confers resistance to 4OHT. (**a**) Western blot analysis of COPS5 expression in MCF7 cell lines stably engineered with empty vector (EV), WT or D151N COPS5 cDNA. (**b**) Quantitative RT–PCR analysis of ERα and its target genes *pS2* and *GREB1* by ERα ligand treatment (48 h) in MCF7 cell lines stably engineered with empty vector (EV), WT or D151N COPS5 cDNA. Data are presented as mean+s.e.m. of three biological replicates. E2: 1 nM, 4OHT: 500 nM. *Significant difference compared with basal (untreated) levels (*P*<0.01, Student's *t*-test). (**c**) Growth of MCF7 cell lines stably engineered with empty vector (Par), WT or D151N COPS5 cDNA in the absence or presence of 4OHT (1 μM). Data are presented as mean+s.e.m. of three biological replicates. *Significant difference compared with other samples (*P*<0.01, Student's *t*-test).

To test if overexpression of COPS5 plays a causative role in driving resistance to tamoxifen, we next established MCF7 cell lines stably expressing wild-type (WT) and metalloprotease-deficient D151N COPS5 (refs 20, 21) by lentiviral infection (Fig. 2a). Of note, ERα expression was not altered by either WT or D151N COPS5 expression (Fig. 2a). In the absence of additional estradiol in culture medium containing full foetal bovine serum (FBS), overexpression of COPS5 did not change the basal expression of ERα target genes *pS2* (*TFF1*) and *GREB1* (Fig. 2b, left four panels). With addition of E2, the expression of both target genes was induced by four to sixfold, while WT COPS5 only marginally induced expression of these genes (Fig. 2b, middle four panels). In the presence of 4OHT, while both *pS2* and *GREB1* were suppressed in parental, vector control and D151N COPS5-overexpressing cell lines, overexpression of WT COPS5 significantly increased the target gene expression (Fig. 2b, right four panels, Student's *t*-test *P*<0.01), indicating that overexpression of COPS5, depending on its catalytic activity, can convert the antagonistic 4OHT to an ERα agonist in breast cancer cells, a hallmark feature of endocrine-resistance at molecular level^11^. Interestingly, the mRNA expression of ERα was not altered by COPS5, suggesting that regulation of ERα target gene expression is likely through a coregulator-related mechanism (Fig. 2b, white bars). In addition to *pS2* and *GREB1*, we examined five more known ERα target genes: *PGR* which is activated by E2 but inhibited by 4OHT in parental MCF7; *NRIP*, *IGFBP4*, *CTSD* for which E2 acts as an agonist while 4OHT acts as a weak agonist as well, and *BMP7* for which E2 is an antagonist. Interestingly, 4OHT strongly induced all target genes except *BMP7* by overexpression of COPS5-WT, but not D151N (Supplementary Fig. 4). These data support the generality of the role of COPS5 amplification/overexpression in switching 4OHT from a SERM to a pure agonist. We further investigated the cell growth phenotype of these MCF7 cell lines. In the absence of 4OHT, WT or D151N COPS5-overexpressing cells did not demonstrate any difference in growth rate *in vitro* compared with the parental cells. However, WT COPS5 cells were still able to proliferate on treatment with 4OHT, whereas parental and D151N-overexpressed cells were inhibited by the antagonist (Fig. 2c). Similarly, in another ERα+ breast cancer cell line T47D, we observed that overexpression of WT but not D151N COPS5 led to agonistic conversion of 4OHT in ERα-dependent transcription (Supplementary Fig. 5a,b), and caused resistance to 4OHT-induced growth inhibition (Supplementary Fig. 5c). Together, these data support that amplification and overexpression of COPS5 in ERα+ breast cancer cells confers resistance to tamoxifen.

### COP9 complex induces tamoxifen-resistance by degrading NCoR

**Figure 3:**
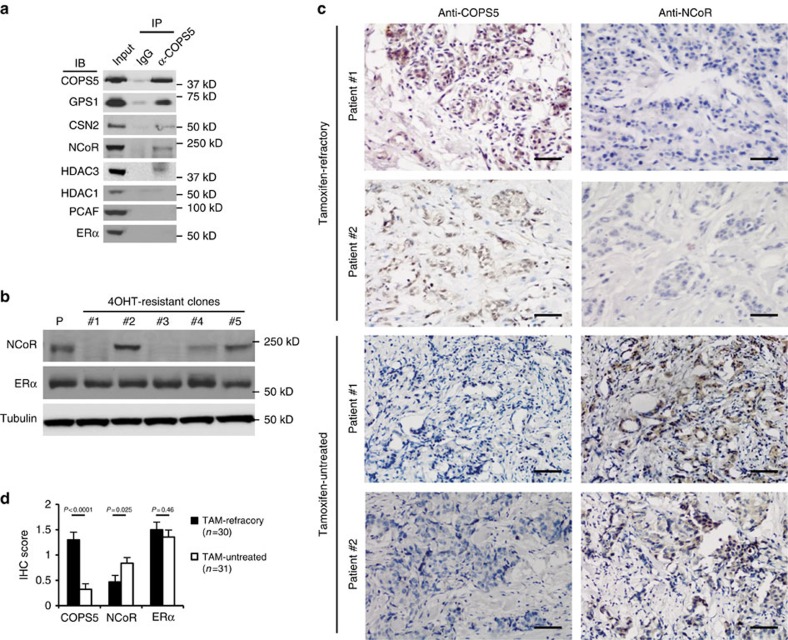
NCoR protein is downregulated in COPS5-overexpressed breast cancer cells. (**a**) Western blot analysis of proteins that were co-immunoprecipitated with anti-COPS5 antibodies in MCF7 parental cells. (**b**) Western blot analysis of NCoR and ERα expression in parental and tamoxifen-resistant MCF7 clones. P, parental cell line. (**c**) IHC of NCoR and COPS5 expression in representative tamoxifen-untreated and refractory breast cancer samples (scale bar, 50 μm). Anti-COPS5 and anti-NCoR antibodies showed nuclear and nuclear/cytoplasmic staining in tumor cells, respectively. (**d**) Quantification and statistical analysis of IHC staining of COPS5, NCoR and ERα in tamoxifen-untreated and refractory breast cancer samples.

Next we sought to elucidate the mechanisms underlying COPS5-mediated tamoxifen-resistance. The Zn^2+^-dependent isopeptidase/deneddylase COPS5 is the catalytic component of the COP9 signalosome complex which is involved in regulating the activity of Skp, Cullin, F-box containing (SCF) E3 ubiquitin ligases and subsequently the stability of substrates^22^,^23^. To explore the potential targets of COPS5 in ERα-regulated transcription, we performed co-immunoprecipitation experiments in tamoxifen-sensitive parental MCF7 cells. We confirmed that COPS5 co-existed with other COP9 components, for example, GPS1/CSN1 and CSN2 (Fig. 3a). Intriguingly, while ERα, coactivator PCAF and corepressor HDAC1 did not appear to associate with COPS5/COP9, the NCoR corepressor complex, including NCoR and HDAC3, was effectively pulled down with the COP9 subunits (Fig. 3a). Reciprocally, immuneprecipitation of endogenous NCoR also pulled down COPS5 (Supplementary Fig. 6a). Indeed, we observed a remarkable reduction of NCoR protein expression in the tamoxifen-resistant MCF7 clones (#1 and #3) in which COPS5 is overexpressed (Figs 1i and 3b), suggesting a functional interaction between COP9 and NCoR complex. Of note, ERα has been proposed to be destabilized by COP9/COPS5 in an agonist-dependent manner^24^. However, in the tamoxifen-resistance MCF7 cell lines, we did not detect genomic alterations of the *ESR1* gene which encodes ERα, and ERα protein was expressed at similar levels in these clones compared with parental MCF7 cells (Fig. 3b). We also investigated tamoxifen-naive (*n*=31) and refractory (*n*=30) breast tumour samples used in Fig. 1, and observed an inverse correlation between NCoR and COPS5 protein levels by IHC staining (Fig. 3c). We then quantified the IHC staining for COPS5, NCoR and ERα (Supplementary Table 2). Intriguingly, while COPS5 expressed significantly higher in refractory tumours (Pearson's *χ*^2^-test *P*<0.0001), it is also significant that NCoR expression is decreased in tamoxifen-resistant breast tumours (Pearson's *χ*^2^-test *P*=0.025) (Fig. 3d). In contrast, ERα expression showed no significant difference between tamoxifen-naive and refractory groups (Pearson's *χ*^2^-test *P*=0.46), suggesting that ERα expression is not a key downstream event of COPS5 overexpression (Fig. 3d, Supplementary Table 2).

**Figure 4:**
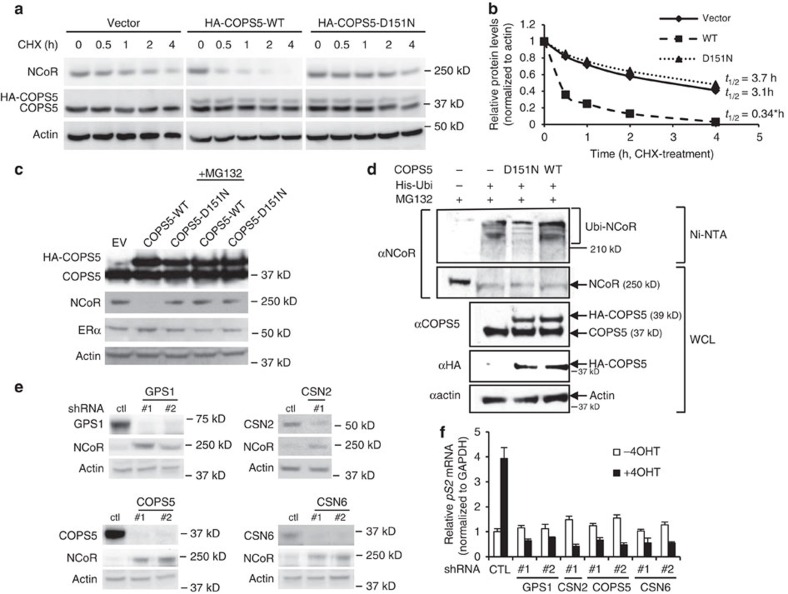
COPS5 overexpression leads to degradation of NCoR protein through the ubiquitin–proteasome pathway. (**a**) Cycloheximide (CHX) chase assay in parental MCF7 cells transduced with vector, HA-COPS5-WT or HA-COPS5-D151N. Cells were treated with CHX and then analysed by western blotting. (**b**) Half-life of NCoR protein was calculated by quantification of the western blots of anti-NCoR and anti-actin (for normalization). *Significant difference compared with other samples (*P*<0.05, Student's *t*-test). (**c**) Western blot analysis of COPS5, NCoR and ERα in MCF7 cells stably expressing empty vector (EV), WT or D151N COPS5 cDNA in the presence or absence of proteasome inhibitor MG132. (**d**) *In vivo* ubiquitination assay in HEK293T cells with overexpression of WT or D151N COPS5. Proteins pulled down by Ni-NTA beads and existed in whole cell lysates (WCL) are tested by western blot using antibodies shown on the left. His-Ubi, His_6_-tagged ubiquitin; Ubi-NCoR, polyubiquitinated NCoR protein. (**e**) Western blot analysis of NCoR expression on knockdown of COP9 subunits GPS1, CSN2 and CSN6 by specific shRNAs in 4OHT-resistant MCF7 clone #3. Ctl, control. (**f**) Quantitative RT–PCR of mRNA of ERα target gene *pS2* in the MCF7 cell lines shown in **e** in response to 4OHT (500 nM) for 48 h. Data are presented as mean+s.e.m. of three biological replicates. All of the 4OHT-treated samples are significantly different from corresponding untreated samples (*P*<0.01, Student's *t*-test).

We then hypothesized that COPS5 regulated the protein stability of NCoR through COP9-mediated regulation ubiquitination/proteasomal activity. First we performed a cycloheximide (CHX) chase assay to measure the half-life of NCoR in the absence and presence of COPS5 overexpression (Fig. 4a). While half-life of NCoR protein is ∼3.1 h in parental MCF7 cells, overexpression of COPS5-WT significantly reduced the half-life to 0.34 h (Fig. 4a,b). In contrast, overexpression of the isopeptidase-deficient COPS5-D151N did not alter the half-life of NCoR (3.7 h) substantially (Fig. 4a,b), suggesting the importance of the catalytic activity. Next, we noticed that NCoR protein, rather than mRNA, was downregulated by overexpression of WT COPS5 in parental MCF7 cells (Fig. 4c, Supplementary Fig. 6b). In the presence of proteasome inhibitor MG132, NCoR protein expression was restored to basal level (Fig. 4c). Again, the ERα protein level was not changed in these cells (Fig. 4c). We then conducted *in vivo* ubiquitination assays by which we demonstrated that overexpression of WT COPS5 augmented ubiquitination of NCoR while the D151N mutant partially antagonized NCoR ubiquitination (Fig. 4d), presumably due to the dominant negative function^20^. Interestingly, both K63R and K48R ubiquitins reduced the overall ubiquitination levels, suggesting that both K63 and K48 are involved in poly-ubiquitination of NCoR (Supplementary Fig. 7). However, COPS5-WT only induced K63R-ubiquitination but lost its activity on K48R-ubiquitination, indicating that K48 is the major lysine residue which COPS5 regulates, though the E3 ligase(s) is yet to be identified (Supplementary Fig. 7). These data are in line with extensive evidence in the literature that proteasomal degradation-related poly-ubiquitination is mainly linked to ubiquitin-K48. To further evaluate the role of COP9 complex in COPS5-mediated NCoR protein degradation, we examined the role of some COP9 subunits by RNAi. In COPS5-overexpressing tamoxifen-resistant MCF7 clone #3, RNAi-mediated knockdown of GPS1/CSN1 (two independent short hairpin RNAs (shRNAs)), CSN2 (one shRNA), COPS5 (two independent shRNAs) or CSN6 (two independent shRNAs) invariably caused induction of NCoR protein expression (Fig. 4e). Moreover, knockdown of these COP9 subunits led to restoration of tamoxifen-dependent transcriptional repression (Fig. 4f, Supplementary Fig. 8). Last, overexpression of WT, but not D151N, COPS5 decreased the levels of neddylated cullins in parental MCF7, whereas RNAi-mediated knockdown of COPS5 induced the neddylation of cullins in COPS5-overexpressed 4OHT-resistant MCF7 clone #3 (Supplementary Fig. 9). These data indicate that COPS5 regulates neddylation of E3 ligase-associated cullins in breast cancer cells. Taken together, it is conceivable that mechanistically overexpression of COPS5 leads to degradation of NCoR protein through the ubiquitination-proteasomal pathway mediated by the COP9 complex in tamoxifen-resistant ERα+ breast cancer cells.

**Figure 5:**
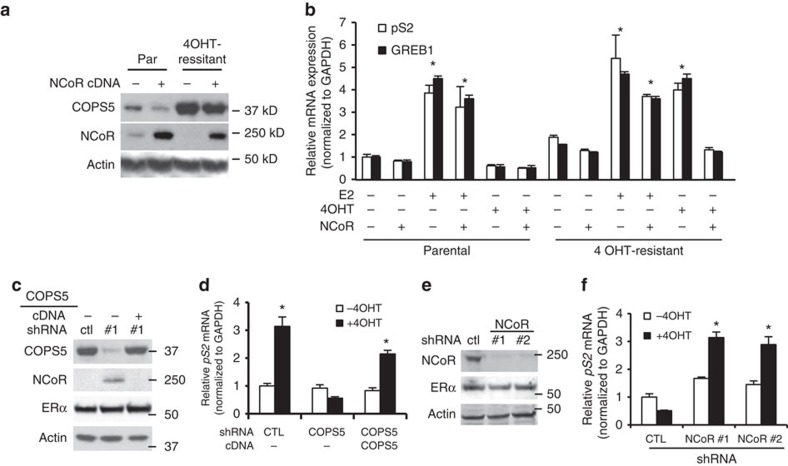
COPS5-mediated 4OHT-resistance is dependent on NCoR downregulation. (**a**) Western blot analysis of COPS5 and NCoR in MCF7 cells with transient expression of COPS5 and/or NCoR. (**b**) Quantitative RT–PCR analysis of ligand-regulated ERα target gene expression in parental and 4OHT-resistant MCF7 clones with expression of NCoR. *Significant difference compared with corresponding untreated basal levels (*P*<0.01, Student's *t*-test). (**c**) Western blot analysis of COPS5, NCoR and ERα expression in the 4OHT-resistant MCF7 cells stably transduced with COPS5 shRNA or rescuing COPS5 cDNA. (**d**) Quantitative RT–PCR analysis of ERα target gene *pS2* in the MCF7 cell lines shown in **c** in response to 4OHT (500 nM, 48 h). *Significant difference compared with the untreated CTL sample (*P*<0.01, Student's *t*-test). (**e**) Western blot analysis of NCoR and ERα expression on knockdown by stable NCoR shRNAs in parental MCF7 cells. (**f**) Quantitative RT–PCR analysis of ERα target gene *pS2* in the MCF7 cell lines shown in **e** in response to 4OHT (500 nM, 48 h). *Significant difference compared with the untreated CTL sample (*P*<0.01, Student's *t*-test). Data are presented as mean+s.e.m. of three biological replicates. CTL, control.

Next we questioned if degradation of NCoR acts as the key step by which amplification or overexpression of COPS5 causes refractory to tamoxifen-treatment. To this end, we overexpressed NCoR complementary DNA (cDNA) in both parental and 4OHT-resistant MCF7 clone #3. Apparently, transgenic overexpression of NCoR largely overcame the COPS5-mediated degradation and restored the NCoR protein level in clone #3 (Fig. 5a). While E2 displayed a similar agonism in both parental and resistant clone #3, 4OHT showed a robust agonistic activity on expression of five ERα target genes (*pS2*, *GREB1*, *PGR*, *NRIP* and *IGFBP4*) selectively in clone #3 (Fig. 5b, Supplementary Fig. 10). Strikingly, re-expression of NCoR in clone #3 restored the antagonistic 4OHT-mediated suppression of all tested ERα target genes (Fig. 5b, last two lanes, Supplementary Fig. 10). Knockdown of COPS5 led to induction of NCoR (but not ERα) in clone #3, which is antagonized by re-expression of exogenous COPS5 cDNA that was designed to avoid the recognition by shRNA, indicating that the shRNA is highly specific to COPS5 (Fig. 5c). Furthermore, knockdown of COPS5 restored the antagonistic/weak agonistic activity of 4OHT in clone #3, whereas re-overexpression of COPS5 abolished the therapeutically beneficial activity again, using expression of five ERα target genes (*pS2, GREB1, NRIP, IGFBP4* and *CTSD*) as biomarkers of ERα-dependent transcription (Fig. 5d, Supplementary Fig. 11). Last, we knocked down NCoR in parental MCF7 cells by two independent shRNAs, which did not impact the expression of ERα (Fig. 5e). Loss of NCoR strongly activated ERα target gene expression (*pS2, GREB1, NRIP, IGFBP4* and *CTSD*) on 4OHT-treatment, mimicking the resistance features observed in the spontaneous tamoxifen-resistant clones and engineered COPS5-overexpressing cell lines (Fig. 5f, Supplementary Fig. 12). Notably, in TCGA ERα+ breast cancer, deleterious mutations and deep deletion of *NCOR1*, the gene coding NCoR, were observed (Supplementary Fig. 13). And it has been shown that the NCoR/HDAC corepressor is required for 4OHT-mediated ERα-dependent transcriptional repression^25^,^26^,^27^. These data strongly suggest that loss of NCoR protein expression by COPS5 amplification and overexpression is a driver of mediating tamoxifen-resistance.

**Figure 6:**
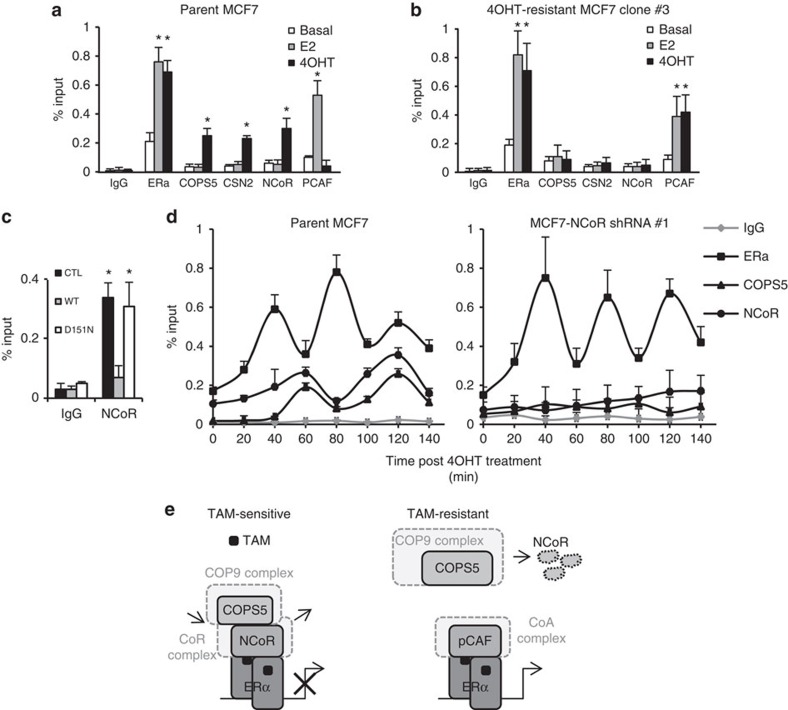
COPS5 overexpression alters the dynamics of coregulator exchange on the *pS2* promoter. ChIP analysis of the *pS2* promoter occupancy of ERα, COPS5, CSN2, NCoR and PCAF on stimulation with ERα ligands for 2 h in (**a**) parental MCF7 cells and (**b**) 4OHT-resistant MCF7 clone #3. E2, 1 nM; 4OHT, 1 μM. Data are presented as mean+s.e.m. from three independent experiments. *Significant difference compared with corresponding basal recruitment (*P*<0.01, Student's *t*-test). (**c**) ChIP analysis of NCoR on stimulation with ERα ligands for 2 h in parental and engineered COPS5-overexpression MCF7 cells. *Significant difference compared with corresponding basal recruitment (*P*<0.01, Student's *t*-test). (**d**) Kinetic ChIP analysis of ERα, COPS5 and NCoR on the *pS2* promoter at 20-min interval in parental MCF7 or NCoR shRNA #1 cells. Data are presented as mean+s.d. from two independent experiments. (**e**) A schematic representation of the proposed molecular model of functional interactions between COPS5 and NCoR in ERα-dependent transcription based on the ChIP data (see text for details). TAM, tamoxifen.

To further understand the molecular mechanisms of COPS5/COP9 complex-mediated regulation of ERα-dependent transcription, we conducted chromatin immunoprecipitation (ChIP) experiments on the *pS2* gene promoter region surrounding the oestrogen-responsive elements (ERE). At basal conditions there was only minimal promoter occupancy of ERα, corepressor NCoR, coactivator PCAF, and COP9 subunits COPS5 and CSN2 in both parental and 4OHT-resistant MCF7 cells (Fig. 6a,b, white bars). In the presence of E2, ERα and PCAF bound to the *pS2* promoter as shown previously^28^,^29^. By contrast, COP9 subunits (COPS5/CSN2) and NCoR were not recruited (Fig. 6a,b, grey bars), indicating that COP9 and NCoR are not directly involved in transcriptional activation by promoter modulation. Surprisingly, on treatment with antagonist 4OHT in parental MCF7 cells, ERα, NCoR, COPS5 and CSN2, but not PCAF, coincidentally occupied the *pS2* promoter, which suggests that basal level of COP9 may directly regulate NCoR on the promoter. However, in 4OHT-resistant MCF7 cells, neither NCoR nor COP9 was recruited, whereas ERα and PCAF were able to bind to the promoter on 4OHT-treatment (Fig. 6a,b, black bars), due probably to global loss of NCoR by overexpression of COPS5. We also confirmed a loss of NCoR recruitment on the *pS2* promoter on engineered overexpression (Fig. 4c) of WT but not D151N COPS5 in MCF7 cells (Fig. 6c). We further performed kinetic ChIP experiments, which revealed that there was a cyclical sequential promoter recruitment of ERα, NCoR and COPS5 after 4OHT-treatment in parental MCF7 cells (Fig. 6d, left panel). Interestingly, the on/off cycles of ERα and NCoR/COPS5 are operated at different rate, which would suggest that COPS5 occupancy is associated with NCoR rather than ERα. Indeed, when we knocked down NCoR by RNAi in parental MCF7 cells, the ERα cyclical recruitment was not impacted whereas the recruitment of COPS5 was completely abolished (Fig. 6d, right panel). This also indicates that promoter occupancy of basal COP9/COPS5 is NCoR-dependent, supporting the protein interaction and gene expression data shown above. In summary, based on the ChIP data, we proposed a model mechanism by which NCoR and COPS5 functionally interact in orchestrating the ERα-dependent transcription: (1) in tamoxifen-sensitive breast cancer cells, tamoxifen triggers the recruitment of ERα/NCoR corepressor transcriptional apparatus. Low basal level of COP9/COPS5 is associated with NCoR and constantly degrade/recycle the NCoR complex as part of the repression strategy to prevent coactivator recruitment, similar to the mechanism of cyclical activation^28^,^29^. Therefore, tamoxifen retains as an antagonist; (2) in tamoxifen-resistant cells, NCoR is globally diminished by degradation due to COPS5 overexpression and consequently high levels of COP9/COPS5 activity. In the absence of NCoR, COP9/COPS5 is no longer recruited by tamoxifen-treatment. Instead, the coactivator complex (PCAF as tested) occupies the ERα-dependent transcriptional promoters/enhancers, leading to drug resistance via a conversion of 4OHT to an agonist (Fig. 6e).

### Inhibition of COPS5 restores tamoxifen sensitivity

**Figure 7:**
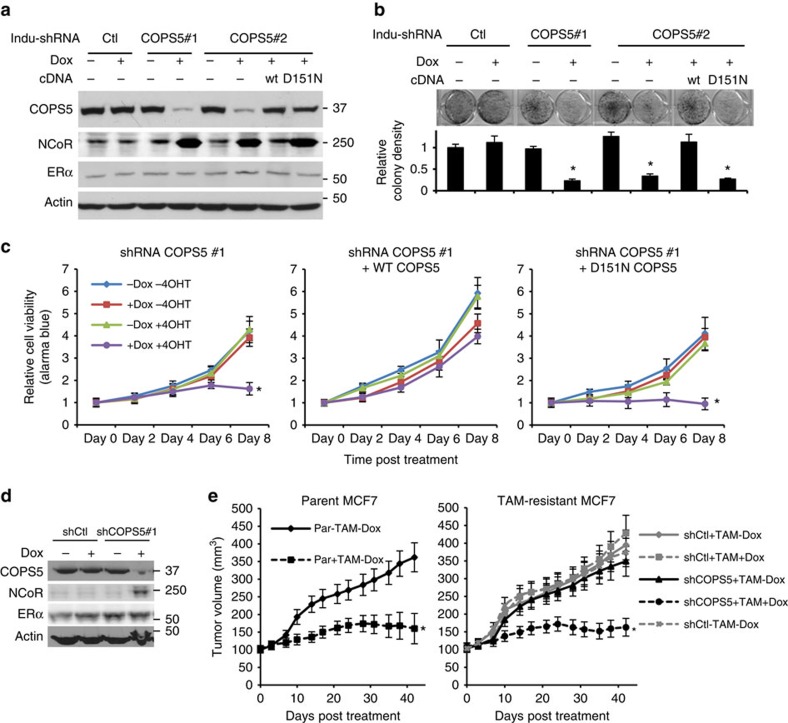
Genetic inhibition of COPS5 re-sensitizes tamoxifen-resistant breast cancer cells to endocrine therapy. (**a**) Western blot analysis of doxycycline (Dox)-inducible shRNA-mediated knockdown of COPS5 and complementary re-expression of COPS5 cDNAs in the cultured 4OHT-resistant MCF7 clone #1. (**b**) Colony formation assay of 4OHT-resistant MCF7 clone #1 on knockdown by COPS5 shRNAs and rescue by COPS5 cDNAs after 4OHT-treatment for 14 days at 1 μM. *Significant difference compared with basal growth (*P*<0.01, Student's *t*-test). (**c**) Growth of 4OHT-resistant MCF7 clone #3 in response to COPS5 knockdown and 4OHT-treatment (1 μM). Data are presented as mean+s.e.m. from three independent experiments. *Significant difference compared with other samples (*P*<0.01, Student's *t*-test). (**d**) Western blot analysis of the xenograft tumours derived from 4OHT-resistant MCF7 clone #3. Cells were stably engineered with doxycycline-inducible shRNAs. (**e**) Volumes of the MCF7 xenograft tumours on treatments as indicated. Data are presented as mean±s.d. of five mice each group. *Significant difference compared with other samples (*P*<0.01, Student's *t*-test). Ctl, control; DOX, doxycycline; Indu-shRNA, inducible shRNA; Par, parental; TAM, tamoxifen.

Finally, we investigated the cancer biological relevance and therapeutic potentials of COPS5-related molecular mechanisms in endocrine-refractory breast cancer. Using the doxycycline-inducible shRNA system, we established stable cell lines with shRNAs targeting *COPS5* and *COPS5* cDNAs in 4OHT-resistant MCF7 cells (Fig. 7a). When COPS5 was knocked down by RNAi, an upregulation of NCoR expression can be clearly observed as we showed above (Figs 4e, 5c and 7a). Crucially, COPS5-WT but not D151N was able to abolish the shRNA-mediated upregulation of NCoR (Fig. 7a), confirming the importance of catalytic activity of COPS5 in regulating NCoR protein expression. ERα protein expression was not affected by any of these genetic modulations (Fig. 7a). Moreover, these cells were proliferating similarly with or without 4OHT-treatment when COPS5 was maintained at high level using control shRNA. By contrast, knockdown of COPS5 by two independent shRNAs re-sensitized the cells to tamoxifen-treatment. Importantly, expression of a shRNA-resistant WT *COPS5* cDNA was able to completely rescue the growth inhibition induced by shRNA, while the isopeptidase-deficient D151N failed to do so (Fig. 7b), indicating that the catalytic activity of COPS5 is essential to confer tamoxifen-resistance in breast cancer cells. In time-course studies, COPS5 shRNA exhibited growth inhibition of the resistant cells only in the presence of 4OHT. Again, this phenotype was rescued by WT COPS5 but not the D151N mutant (Fig. 7c). It is therefore conceivable that combination of tamoxifen and a COPS5 inhibitor would be a desired therapeutic approach to overcoming tamoxifen-resistance in breast cancer. Last, we tested the *in vivo* role of COPS5 in mediating tamoxifen-refractory. Using the MCF7 xenograft model, we first confirmed that induction of COPS5 shRNA, but not the control shRNA, led to substantial knockdown of COPS5 expression after doxycycline-treatment *in vivo* (Fig. 7d). Subsequently, only NCoR expression was upregulated whereas ERα did not change (Fig. 7d). As expected, the tumours derived from parental MCF7 cells were sensitive to tamoxifen-treatment, as shown extensively in the literature. Dosing tamoxifen at 100 mg kg^−1^ three times per week induced a substantial stasis of tumour growth (Fig. 7e). The tumours derived from the 4OHT-resisatant MCF7 clone #3 were completely resistant to the same regimen though the basal growth (vehicle-only) was similar to the parental tumours. However, knockdown of COPS5 by inducible shRNA restored the sensitivity to tamoxifen-treatment in the COPS5-overexpressed resistant tumours (Fig. 7e). Satisfyingly, COPS5 reduction and subsequent NCoR upregulation by COPS5 shRNA were observed at the end point of doxycycline/tamoxifen-treatment at day 42 *in vivo* (Supplementary Fig. 14, last two lanes). Furthermore, ERα expression was not changed in these MCF7 tumour samples (Supplementary Fig. 14). Interestingly, while knockdown of an essential gene *PLK1* induced a remarkable growth inhibition of the subcutaneous tumours of parental MCF7 cells, neither knockdown nor overexpression of *COPS5* showed a significant impact on MCF7 tumour growth (Supplementary Fig. 15). It suggests that the dependence on COPS5 is selective in COPS5-amplified/overexpressed MCF7 cells on tamoxifen-treatment. These results support that COPS5 is a therapeutic target in COPS5-amplified, tamoxifen-resistant breast cancer.

## Discussion

Drug resistance represents a major clinical challenge in targeted therapy for cancer^30^,^31^. In ERα-positive breast cancer, although most of the patients initially responded well to tamoxifen or other endocrine treatment, eventually many of them inevitably developed to drug-resistant and metastatic disease which is incurable^8^. Although a number of resistance mechanisms have been proposed, most of them failed to translate into clinical benefits to overcoming the resistance. In this report, we revealed a novel genomics-driven mechanism that renders resistance to tamoxifen, namely amplification and overexpression of the gene encoding COPS5, a component of the neddylation-catalyzing COP9 signalosome complex regulating the activity of SCF E3 ligases. In the TCGA primary tumour cohort, amplification of *COPS5* is relatively focal and correlates with high *COPS5* mRNA expression (Fig. 1). It is also evident that overexpression of COPS5 is further enriched in tamoxifen-resistant tumours (Fig. 1). Since (1) the tamoxifen-refractory tumour samples used for IHC were biopsied/autopsied from patients who had been treated with tamoxifen for at least 5 months to >10 years; and (2) short-term treatment of MCF7 cells with tamoxifen *in vitro* or *in vivo* did not alter the COPS5 protein expression (Supplementary Fig. 16), the elevated COPS5 expression is probably due to drug resistance-associated selection (genomic amplification) rather than acute induction by tamoxifen. Interestingly, overexpression of COPS5 induces ubiquitination and proteasomal degradation of the NCoR protein, a key corepressor of tamoxifen-bound ERα, leading to de-repression of ERα target genes in resistant breast cancer (Figs 2, 3, 4, 5). Based on the data we showed in this report, in hormone-sensitive ERα-positive breast cancer normal basal levels of COPS5/COP9 precisely control the protein stability of ERα corepressor NCoR. In the presence of antiestrogen drugs, COP9/COPS5 is associated with NCoR on the target gene promoter and constantly degrades and recycles the NCoR complex as a molecular strategy of transcriptional repression, which is similar to the previously reported mechanism of ERα activation through the cyclical clearance of coregulators^28^,^29^. Therefore, the drugs are able to suppress ERα-regulated transcription and consequently inhibit cancer cell growth. However, when COPS5 is amplified and overexpressed in endocrine-resistant cancer cells, elevated COPS5/COP9 activity induces global NCoR protein degradation. As a result, tamoxifen recruits the coactivator complex (PCAF as tested) instead to the ERα-dependent transcriptional promoters/enhancers, leading to drug resistance via a conversion of tamoxifen to an agonist (Fig. 6e).

The functional relevance of NCoR is further supported by the fact that NCoR is deleteriously mutated in about 5% of ERα-positive breast cancer (TCGA, Supplementary Fig. 13)^32^,^33^. Furthermore, downregulation of NCoR mRNA expression in breast cancer patients correlates with shorter relapse^34^, suggesting its crucial role in breast cancer development. More importantly, we showed that loss of NCoR expression is sufficient to trigger the resistance to tamoxifen, in line with the previous reports. We revealed a switch of transcription coregulators on the promoters of ERα target genes on overexpression of COPS5 and loss of NCoR (Fig. 6e). NCoR is replaced by coactivators including PCAF by tamoxifen-treatment in endocrine-resistant breast cancer cells, leading to activation of the ERα signalling which is required for the cancer cell growth. In fact, a number of endocrine-resistance mechanisms may involve the ERα-dependent transcription, for example, hotspot mutations in ERα ligand-binding domain, NCOA1 amplification, deleterious mutations in NCoR and PI3K activation. This ERα-centric resistance mechanism implicates a possible crosstalk between COPS5 amplification with other aberrant alterations associated with endocrine-resistance. Here we uncovered the COPS5–NCoR–coactivator axis as a key mediator in conferring the drug resistance. Of note, breast cancer cells with overexpression of COPS5 appeared to be less sensitive to the SERD fulvestrant *in vitro* as well (Supplementary Fig. 2), suggesting that it could be a common resistance mechanism to endocrine therapies. Because ERα should be degraded by fulvestrant, the exact molecular mechanism by which COPS5 can induce fulvestrant-resistance is still ambiguous and currently under investigation.

The COP9 protein complex consists of eight subunits in which COPS5 is the only catalytically active metalloprotease. The complex is well-characterized as a master regulator in ubiquitin-mediated protein degradation^35^. COP9 exerts these activities through regulating cullin-RING ligases (CRLs) and RING-containing ubiquitin ligases that target many proteins for ubiquitin/proteasome-mediated degradation. COPS5-mediated removal of Nedd8 (deneddylation) from cullin is a crucial step for CRLs' activity. COPS5 possesses a JAMM (JAB1/MPN/MOV34) metalloprotease motif (EX_n_HXHX_10_D) that is required for cullin deneddylation. It has been shown that COP9/COPS5 can regulate the stability of a variety of cancer-related proteins including p27, p53, MDM2, c-Jun and key proteins involved in DNA damage repair. Consequently its role in tumorigenesis has been proposed even though there is a lack of clear mechanistic understanding^36^,^37^,^38^. Accumulating evidence suggests critical roles of COPS5 in different stages of breast cancer development. A number of studies have demonstrated that COPS5 is overexpressed in breast cancer^39^,^40^,^41^. The involvement of COPS5 in breast cancer, particularly ERα-negative tumours, appears to be linked to the EGFR and HER2 pathways by mediating downstream signalling that contributes to the progression of tumour growth^40^,^41^. In addition, COPS5 has been shown to be involved in breast cancer initiation by coordinating the epithelial transformation evoked by Myc and Ras in mouse models^42^. It was also proposed that ligand-induced ERα degradation was regulated by COPS5 in hormone-dependent breast cancer cells^24^. Here using integrated genomic and functional approaches, we discovered a key role of COPS5/COP9 in conferring resistance to endocrine therapy. We revealed that NCoR is a substrate of COPS5/COP9 that is crucial in mediating endocrine-resistance in breast cancer. It is conceivable that COPS5 might be involved in multiple stages of breast cancer development by regulating different protein targets via its isopeptidase activity. Further identification and elucidation of the E3 ligase that connects COPS5/COP9 and NCoR are of importance to understand the precise mechanisms underlying endocrine resistance and identify additional druggable molecular targets.

Our findings also offer therapeutic opportunities to overcome tamoxifen-resistance. Since the Zn^2+^-dependent metalloprotease activity appears to be critical in the resistance mechanism (Fig. 7), inhibition of the catalytic activity of COPS5 is an intriguing and promising strategy for drug discovery. Alternatively, based on recent finding on the structure of eight-unit COP9 complex^43^, it is possible to modulate COPS5 activity by tackling the interactions between COP9 subunits, particularly the interaction between COPS5 and COPS6/CSN6 subunits that is crucial for maintaining the isopeptidase activity. The major function of the isopeptidase/metalloprotease activity of COPS5/COP9 is deneddylation of cullin proteins in SCF/CRL ubiquitin ligases to trigger the neddylation cycles for maintaining the ligase activity. Consequently, using inhibitors of Nedd8-activating enzymes, for example, MLN4924, might have an impact on the COPS5/NCoR axis^44^. Last, there are kinases like CK2 and PKD that are associated with the COP9 complex and crucial for its activity^45^. Modulation of the kinase activity, which is generally more feasible for drug discovery, could be an approach to inhibiting the COPS5 activity as well. Regardless how the COPS5 inhibitors will be developed, our data suggest that combination with ERα antagonists is probably needed to re-sensitize the refractory tumours to endocrine therapy. In summary, the current work supports the idea of developing COPS5 inhibitors to treat tamoxifen-resistant ERα+ breast cancer patients harbouring *COPS5* amplification and/or overexpression.

## Methods

### Cell lines and reagents

MCF7 and T47D were purchased from ATCC. HEK293FT was purchased from Life Technologies. Tamoxifen-resistant MCF7 clones were generated by dose-escalation 4OHT-treatment in phenol red-free RPMI1640 culture medium supplied with 10% charcoal-stripped FBS (Life Technologies): 200 nM for 2 weeks, 500 nM for 2 weeks and then 2 μM for 4 weeks. Single clones were picked up under microscope and then expanded in 2 μM 4OHT-containing phenol red-free RPMI1640 culture medium supplied with 10% charcoal-stripped FBS. Antibodies used in this report were: COPS5 (A300-014A, Bethyl Laboratories), NCoR (PA1-844A, Thermo Scientific) for western blot (WB) and IHC, NCoR (sc-1609, Santa Cruz) for IP and ChIP, ERα (SC-543, Santa Cruz) for WB, ERα (61035, Active Motif) for ChIP, GPS1 (ab4535, Abcam), CSN2 (PA1-24686, Thermo Scientific), CSN6 (sc-47965, Santa Cruz), NEDD8 (Y297) (ab81264, Abcam), PCAF (sc-13124, Santa Cruz), HDAC1 (sc-6298, Santa Cruz), HDAC3 (sc-17795, Santa Cruz), HA-tag (MMS-101P, Covance), His-tag (sc-803, Santa Cruz), actin (A-3853, Sigma), tubulin (T6074, Sigma) and secondary antibodies were purchased from Sigma. Compounds used were tamoxifen (T5648, Sigma), 4OHT (94873, Sigma), E2 (E8875, Sigma), fulvestrant (I4409, Sigma), flavopiridol (S2679, Selleckchem) and MG132 (S2619, Selleckchem).

### Lentiviral RNAi and cDNA

The inducible shRNA vector was modified from the previous reports^46^,^47^. Target sequences are listed below: COPS5 #1: 5′- GTCTCAGGTTATTAAGGATAA -3′, COPS5 #2: 5′- CAGTCTCTGAGAAGTACTTTA -3′, NCoR #1: 5′- GCTCTCAAAGTTCAGACTCTT -3′, NCoR #2: 5′- GCCCACAGATGATGAAGAAAT -3′, GPS1 #1: 5′- CCGAGACATCATCTTCAAATT -3′, GPS1 # 2: 5′- CGCTGCCGGTTCAGGTGTTTA -3′, CSN2 #1: 5′- GCACTCTATGAACAGTCACTT -3′, CSN6 #1: 5′- CGTGGAAGAGAAGATTATCAT -3′, CSN6 #2: 5′- CAGTTTGTGAACAAGTTCAAT -3′. Lentiviruses were packaged by co-transfecting 1 × 10^7^ HEK293FT cells with 5 μg of shRNA-encoding plasmid, 1.5 μg each of Δ8.91 packaging plasmid, pCMV-VSV-G envelop plasmids using TransIT-293 (Mirus). Cell culture media was changed after 24 h and lentivirus-containing supernatant was harvested 72 h post transfection. Infection of the target cells was carried out with a final concentration of 8 μg ml^−1^ polybrene-containing viral medium and selected in 1 μg ml^−1^ puromycin (for shRNA or cDNA vector) or 200 μg ml^−1^ neomycin (for cDNA vector) after 48 h post infection.

### WB analysis

Five per cent non-fat milk in TBS-T (0.1% tween 20) was used as blocking and probing buffer. TBS-T was used as wash buffer. Cells were harvested by scraping into ice-cold PBS and lysed using RIPA buffer (150 mM NaCl, 0.5% Triton X-100, 1% sodium deoxycholate, 0.1% SDS, 1 mM EDTA and 50 mM Tris-HCl, pH 7.4) supplemented with protease inhibitor cocktails (Roche). Antibodies were probed using manufacturer's recommended conditions. Signals were detected using the ECL detection system (GE Healthcare). NIH ImageJ software was used for quantification of WBs. Uncropped scans were shown in Supplementary Fig. 17.

### Quantitative RT–PCR

Total RNA was purified using the RNeasy kit (Qiagen), and treated with DNase before cDNA preparation. cDNA was synthesized following manufacturer's manual (SuperScript III RT, Life Technologies). mRNA levels were determined using TaqMan Probe-Based Gene Expression Assays using ABI PRISM Detection System (Applied Biosystems/Life Technologies). All primers and probes of genes tested in this report were purchased from Life Technologies. VIC-MGB GAPDH control primers and probe were examined as internal controls. All samples were tested in triplicate and normalized to GAPDH levels. Gene-specific relative mRNA levels were calculated by the standard equation 2^(ΔCT sample−ΔCT control)^.

### Copy number

TaqMan qPCR copy number assay (Life Technologies) was used to measure the copy numbers of COPS5 in genomic DNA following the protocol from the vendor. LINE1 was used as the internal normalization control.

### Immunoprecipitation

NETN buffer (25 mM Tris-HCl, pH 8.0, 150 mM NaCl, 1 mM EDTA, 1% (v/v) Nonidet P-40, 10% (v/v) glycerol, 1 × protease inhibitor mix) was used for lysis and immunoprecipitation. Immunoprecipitation was performed using standard protocol^48^.

### Chromatin immunoprecipitation

Chromatin extraction (ab117152) and ChIP kits (ab117138) were purchased from Abcam. ChIP was conducted following the instructions of vendor. Primers used for pS2-ERE promoter are: forward: 5′- GCTTAGGCCTAGACGGAATGGGC -3′; reverse: 5′- CCAGGTCCTACTCATATCTGAGAG -3′.

### CHX chase experiment

MCF7 cells are infected with COPS5-WT- or D151N-expressing lentivirus, and 16 h later selected by puromycin (2 μg ml^−1^) for 24 h. The cells were then treated with 20 μg ml^−1^ of CHX for 0.5–4 h, and cell lysates were isolated for WB analysis.

### *In vivo* ubiquitination assay

HEK293T cells were co-transfected with plasmids encoding His6-tagged ubiquitin and NCoR, and harvested 36 h after transfection. The cells were lysed in 6 ml of guanidinine lysis buffer (6 M guanidinium-HCl, 100 mM Na2HPO4/NaH2PO4, 10 mM Tris, pH 8, 5 mM imidazole and 10 mM β-mercaptoethanol). Then 100 μl of Ni2+-nitrilotriacetic acid-agarose beads (Qiagen) was added into cell lysates, followed by incubation overnight. The beads were then washed with the following buffers: guanidinine buffer (6 M guanidinine-HCl, 100 mM Na2HPO4/NaH2PO4, 10 mM Tris-HCl, pH 8, 10 mM β-mercaptoethanol), urea buffer (8 M urea, 100 mM Na2HPO4/NaH2PO4, 10 mM Tris-HCl, pH 8, 10 mM β-mercaptoethanol), buffer A (8 M urea, 100 mM Na2HPO4/NaH2PO4, 10 mM Tris-HCl, pH 6.3, 10 mM β-mercaptoethanol) and buffer A plus 0.1% Triton X-100. Elution was carried out with 200 mM imidazole in 150 mM Tris-HCl (pH 6.7) supplemented with 5% SDS, 30% glycerol and 500 mM β-mercaptoethanol. The eluates were diluted 1:1 with 2 × SDS–PAGE sample buffer and subjected to SDS–PAGE, and ubiquitinated proteins probed with specific antibodies by WB analysis.

### Cell viability assays

*In vitro* cell growth and viability assays were performed by the alamarBlue Cell Viability Assay kit (Life technologies) following the vendor's instruction. Colony formation experiments were conducted using crystal violet staining^49^. Quantification of colony density was measured by dissolving crystal violet using 10% acetic acid for 20 min, and then followed by measurement of optical density at 590 nm.

### Acquisition and analysis of TCGA data

Data were downloaded from the TCGA data portal ( https://tcga-data.nci.nih.gov/tcga/tcgaDownload.jsp) and visualized or analysed by Excel (Microsoft) and/or the cBio visualization portal ( http://www.cbioportal.org/).

### Clinical samples

From January 2005 to December 2014, the tissues of breast cancer patients with tamoxifen-refectory (*n*=30; age range, 21–70 years) and the tissues of tamoxifen-naive breast cancer as the controls (*n*=31; age range, 20–73 years) were collected, respectively. The cohorts were closely matched for age enroled into this study (*P*>0.05). The patients' medical records were retrospectively reviewed to collect diagnostic information. All subjects, enroled in this study at the Fudan University Shanghai Cancer Center (Shanghai, China), gave written informed consent. The Institute Ethical Committee approved the study protocol according to the guidelines of Helsinki conventions.

### Immunohistochemistry

IHC staining for COPS5 and NCoR was performed on formalin-fixed paraffin-embedded tissues using a protocol as previously described^50^. COPS5 was detected with a commercially available rabbit polyclonal antibody at a dilution of 1:200. To evaluate NCoR expression, we used rabbit polyclonal antibody PA1-844A (dilution 1:400). Anti-ERα antibody (dilution 1:500, GeneTex Inc) was used for IHC of ERα. IHC staining was assessed by two independent pathologists under blinded conditions. The concordance between scores from different cores of the same tumour was >90%. The intensity of COPS5 and NCoR staining was evaluated using the following criteria: we regarded close to 100% of cells exhibiting strong nuclear staining as a very strong positive result (+++); >75% of cells exhibiting strong cytoplasmic and/or nuclear staining as a strong positive result (++); and 50–75% of cells exhibiting strong cytoplasmic and/or 10–75% of cells with nuclear staining as a positive result (+), whereas weak cytoplasmic expression (to any extent) in<50% or <10% nuclear staining of cells was considered as negative expression (−)^51^.

### Animal experiments

All animal studies were performed under regulations provided by the Zhejiang Provincial Experimental Animal Regulation Committee and the Shanghai Municipal Experimental Animal Regulation Committee. Ten million MCF7 cells were inoculated s.c. in 4–6-week-old female, intact athymic nude mice. Estradiol pellets (0.5 mg per pellet) were implanted s.c. into the right back skin between the ear and shoulder. When the tumours were palpable (∼100 mm^3^), mice were randomized into treatment groups (five animals per group). For inducing shRNA expression, animals received doxycycline hyclate (40 mg kg^−1^ per day by oral gavage) for the duration of the study. Tamoxifen-treatment (20 mg kg^−1^ of tamoxifen citrate in peanut oil) was administered s.c. daily Monday through Friday for the duration of the study. Vehicles were dosed in control groups. Tumour volume and body weight were measured twice per week. Tumour volume was determined by using calipers for measurement of the longest (Length) and shortest (Width) dimensions and calculated as (Length × Width^2^)/2. To monitor shRNA knockdown efficiency, a separate group of tumour tissues were collected after 2 weeks of doxycycline-treatment and snap-frozen in liquid nitrogen for WB analysis.

### Statistics

Tumour size comparisons between groups were done using one-way analysis of variance. *In vitro* cell growth data, transcription quantitative reverse transcription–PCR (qRT–PCR) data and other biological assay data were compared between two groups using Student's *t*-test (two-tailed). Tumour sample expression data and IHC staining data were analysed by Pearson's *χ*^2^-test. For all tests, the level of significance was set at *P*<0.05.

### Data availability

The authors declare that all other data supporting the findings of this study are available within the article and its Supplementary Information files.

## Additional information

**How to cite this article:** Lu, R. *et al*. *COPS5* amplification and overexpression confers tamoxifen-resistance in ERα-positive breast cancer by degradation of NCoR. *Nat. Commun.* 7:12044 doi: 10.1038/ncomms12044 (2016).

## Supplementary Material

Supplementary Information Supplementary Figures 1-17 and Supplementary Tables 1-2

## References

[b1] Burstein H. J. . Adjuvant endocrine therapy for women with hormone receptor-positive breast cancer: american society of clinical oncology clinical practice guideline focused update. J. Clin. Oncol. 32, 2255–2269 (2014). 24868023 10.1200/JCO.2013.54.2258 PMC4876310

[b2] Charehbili A. . Neoadjuvant hormonal therapy for endocrine sensitive breast cancer: a systematic review. Cancer Treat. Rev. 40, 86–92 (2014). 23891267 10.1016/j.ctrv.2013.06.001

[b3] Lumachi F. . Endocrine therapy of breast cancer. Curr. Med. Chem. 18, 513–522 (2011). 21143113 10.2174/092986711794480177

[b4] Osborne C. K. & Schiff R. Mechanisms of endocrine resistance in breast cancer. Annu. Rev. Med. 62, 233–247 (2011). 20887199 10.1146/annurev-med-070909-182917 PMC3656649

[b5] Normanno N. . Mechanisms of endocrine resistance and novel therapeutic strategies in breast cancer. Endocr. Relat. Cancer 12, 721–747 (2005). 16322319 10.1677/erc.1.00857

[b6] Dees E. C. & Carey L. A. Improving endocrine therapy for breast cancer: it's not that simple. J. Clin. Oncol. 31, 171–173 (2013). 23233714 10.1200/JCO.2012.46.2655

[b7] Arpino G., Wiechmann L., Osborne C. K. & Schiff R. Crosstalk between the estrogen receptor and the HER tyrosine kinase receptor family: molecular mechanism and clinical implications for endocrine therapy resistance. Endocr. Rev. 29, 217–233 (2008). 18216219 10.1210/er.2006-0045 PMC2528847

[b8] Musgrove E. A. & Sutherland R. L. Biological determinants of endocrine resistance in breast cancer. Nat. Rev. Cancer 9, 631–643 (2009). 19701242 10.1038/nrc2713

[b9] Renoir J. M., Bouclier C., Seguin A., Marsaud V. & Sola B. Antioestrogen-mediated cell cycle arrest and apoptosis induction in breast cancer and multiple myeloma cells. J. Mol. Endocrinol. 40, 101–112 (2008). 18316469 10.1677/JME-07-0143

[b10] Conzen S. D. Minireview: nuclear receptors and breast cancer. Mol. Endocrinol. 22, 2215–2228 (2008). 18417735 10.1210/me.2007-0421 PMC2582530

[b11] Naughton C. . Progressive loss of estrogen receptor alpha cofactor recruitment in endocrine resistance. Mol. Endocrinol. 21, 2615–2626 (2007). 17666584 10.1210/me.2007-0110

[b12] Zhang Q. X., Borg A., Wolf D. M., Oesterreich S. & Fuqua S. A. An estrogen receptor mutant with strong hormone-independent activity from a metastatic breast cancer. Cancer Res. 57, 1244–1249 (1997). 9102207

[b13] Toy W. . ESR1 ligand-binding domain mutations in hormone-resistant breast cancer. Nat. Genet. 45, 1439–1445 (2013). 24185512 10.1038/ng.2822 PMC3903423

[b14] Robinson D. R. . Activating ESR1 mutations in hormone-resistant metastatic breast cancer. Nat. Genet. 45, 1446–1451 (2013). 24185510 10.1038/ng.2823 PMC4009946

[b15] Merenbakh-Lamin K. . D538G mutation in estrogen receptor-alpha: A novel mechanism for acquired endocrine resistance in breast cancer. Cancer Res. 73, 6856–6864 (2013). 24217577 10.1158/0008-5472.CAN-13-1197

[b16] Jeselsohn R. . Emergence of constitutively active estrogen receptor-alpha mutations in pretreated advanced estrogen receptor-positive breast cancer. Clin. Cancer Res. 20, 1757–1767 (2014). 24398047 10.1158/1078-0432.CCR-13-2332 PMC3998833

[b17] Clarke R., Tyson J. J. & Dixon J. M. Endocrine resistance in breast cancer—An overview and update. Mol. Cell. Endocrinol. 418, (Pt 3): 220–234 (2015). 26455641 10.1016/j.mce.2015.09.035 PMC4684757

[b18] Beroukhim R. . The landscape of somatic copy-number alteration across human cancers. Nature 463, 899–905 (2010). 20164920 10.1038/nature08822 PMC2826709

[b19] Zack T. I. . Pan-cancer patterns of somatic copy number alteration. Nat. Genet. 45, 1134–1140 (2013). 24071852 10.1038/ng.2760 PMC3966983

[b20] Cope G. A. . Role of predicted metalloprotease motif of Jab1/Csn5 in cleavage of Nedd8 from Cul1. Science 298, 608–611 (2002). 12183637 10.1126/science.1075901

[b21] Peth A., Berndt C., Henke W. & Dubiel W. Downregulation of COP9 signalosome subunits differentially affects the CSN complex and target protein stability. BMC Biochem. 8, 27 (2007). 18093314 10.1186/1471-2091-8-27 PMC2225408

[b22] Cope G. A. & Deshaies R. J. COP9 signalosome: a multifunctional regulator of SCF and other cullin-based ubiquitin ligases. Cell 114, 663–671 (2003). 14505567 10.1016/s0092-8674(03)00722-0

[b23] Wei N. & Deng X. W. The COP9 signalosome. Annu. Rev. Cell Dev. Biol. 19, 261–286 (2003). 14570571 10.1146/annurev.cellbio.19.111301.112449

[b24] Callige M., Kieffer I. & Richard-Foy H. CSN5/Jab1 is involved in ligand-dependent degradation of estrogen receptor {alpha} by the proteasome. Mol. Cell. Biol. 25, 4349–4358 (2005). 15899841 10.1128/MCB.25.11.4349-4358.2005 PMC1140630

[b25] Lavinsky R. M. . Diverse signaling pathways modulate nuclear receptor recruitment of N-CoR and SMRT complexes. Proc. Natl Acad. Sci. USA 95, 2920–2925 (1998). 9501191 10.1073/pnas.95.6.2920 PMC19670

[b26] Jepsen K. . Combinatorial roles of the nuclear receptor corepressor in transcription and development. Cell 102, 753–763 (2000). 11030619 10.1016/s0092-8674(00)00064-7

[b27] Keeton E. K. & Brown M. Cell cycle progression stimulated by tamoxifen-bound estrogen receptor-alpha and promoter-specific effects in breast cancer cells deficient in N-CoR and SMRT. Mol. Endocrinol. 19, 1543–1554 (2005). 15802375 10.1210/me.2004-0395

[b28] Shang Y., Hu X., DiRenzo J., Lazar M. A. & Brown M. Cofactor dynamics and sufficiency in estrogen receptor-regulated transcription. Cell 103, 843–852 (2000). 11136970 10.1016/s0092-8674(00)00188-4

[b29] Metivier R. . Estrogen receptor-alpha directs ordered, cyclical, and combinatorial recruitment of cofactors on a natural target promoter. Cell 115, 751–763 (2003). 14675539 10.1016/s0092-8674(03)00934-6

[b30] Garraway L. A. & Janne P. A. Circumventing cancer drug resistance in the era of personalized medicine. Cancer Discov. 2, 214–226 (2012). 22585993 10.1158/2159-8290.CD-12-0012

[b31] Holohan C., Van Schaeybroeck S., Longley D. B. & Johnston P. G. Cancer drug resistance: an evolving paradigm. Nat. Rev. Cancer 13, 714–726 (2013). 24060863 10.1038/nrc3599

[b32] Stephens P. J. . The landscape of cancer genes and mutational processes in breast cancer. Nature 486, 400–404 (2012). 22722201 10.1038/nature11017 PMC3428862

[b33] Ciriello G. . The molecular diversity of Luminal A breast tumours. Breast Cancer Res. Treat. 141, 409–420 (2013). 24096568 10.1007/s10549-013-2699-3 PMC3824397

[b34] Girault I. . Expression analysis of estrogen receptor alpha coregulators in breast carcinoma: evidence that NCOR1 expression is predictive of the response to tamoxifen. Clin. Cancer Res. 9, 1259–1266 (2003). 12684393

[b35] Wolf D. A., Zhou C. & Wee S. The COP9 signalosome: an assembly and maintenance platform for cullin ubiquitin ligases? Nat. Cell Biol. 5, 1029–1033 (2003). 14647295 10.1038/ncb1203-1029

[b36] Shackleford T. J. & Claret F. X. JAB1/CSN5: a new player in cell cycle control and cancer. Cell Div. 5, 26 (2010). 20955608 10.1186/1747-1028-5-26 PMC2976740

[b37] Lee M. H., Zhao R., Phan L. & Yeung S. C. Roles of COP9 signalosome in cancer. Cell Cycle 10, 3057–3066 (2011). 21876386 10.4161/cc.10.18.17320 PMC3218617

[b38] Pan Y., Yang H. & Claret F. X. Emerging roles of Jab1/CSN5 in DNA damage response, DNA repair, and cancer. Cancer Biol. Ther. 15, 256–262 (2014). 24495954 10.4161/cbt.27823 PMC3974825

[b39] Kouvaraki M. A. . Jun activation domain-binding protein 1 expression in breast cancer inversely correlates with the cell cycle inhibitor p27(Kip1). Cancer Res. 63, 2977–2981 (2003). 12782606

[b40] Wang J. . Jab1 is a target of EGFR signaling in ERalpha-negative breast cancer. Breast Cancer Res. 10, R51 (2008). 18534028 10.1186/bcr2105 PMC2481501

[b41] Hsu M. C. . Jab1 is overexpressed in human breast cancer and is a downstream target for HER-2/neu. Mod. Pathol. 21, 609–616 (2008). 18246048 10.1038/modpathol.2008.23

[b42] Adler A. S. . CSN5 isopeptidase activity links COP9 signalosome activation to breast cancer progression. Cancer Res. 68, 506–515 (2008). 18199546 10.1158/0008-5472.CAN-07-3060 PMC2646416

[b43] Lingaraju G. M. . Crystal structure of the human COP9 signalosome. Nature 512, 161–165 (2014). 25043011 10.1038/nature13566

[b44] Soucy T. A. . An inhibitor of NEDD8-activating enzyme as a new approach to treat cancer. Nature 458, 732–736 (2009). 19360080 10.1038/nature07884

[b45] Uhle S. . Protein kinase CK2 and protein kinase D are associated with the COP9 signalosome. EMBO J. 22, 1302–1312 (2003). 12628923 10.1093/emboj/cdg127 PMC151059

[b46] Meerbrey K. L. . The pINDUCER lentiviral toolkit for inducible RNA interference *in vitro* and *in vivo*. Proc. Natl Acad. Sci. USA 108, 3665–3670 (2011). 21307310 10.1073/pnas.1019736108 PMC3048138

[b47] Wiederschain D. . Single-vector inducible lentiviral RNAi system for oncology target validation. Cell Cycle 8, 498–504 (2009). 19177017 10.4161/cc.8.3.7701

[b48] Ma Y. . Histone deacetylase 3 inhibits new tumour suppressor gene DTWD1 in gastric cancer. Am. J. Cancer Res. 5, 663–673 (2015). 25973305 PMC4396045

[b49] Franken N. A., Rodermond H. M., Stap J., Haveman J. & van Bree C. Clonogenic assay of cells *in vitro*. Nat. Protoc. 1, 2315–2319 (2006). 17406473 10.1038/nprot.2006.339

[b50] Lu R., Sun M., Feng J., Gao X. & Guo L. Myofibrillogenesis regulator 1 (MR-1) is a novel biomarker and potential therapeutic target for human ovarian cancer. BMC Cancer 11, 270 (2011). 21702971 10.1186/1471-2407-11-270 PMC3132198

[b51] Lu R. . Lactate dehydrogenase 5 expression in Non-Hodgkin lymphoma is associated with the induced hypoxia regulated protein and poor prognosis. PLoS ONE 8, e74853 (2013). 24086384 10.1371/journal.pone.0074853 PMC3781153

