# Supplementary material for: COPS5 amplification and overexpression confers tamoxifen-resistance in ERα-positive breast cancer by degradation of NCoR
Source: Nat Commun. 2016 Jul 4;7:12044. doi: 10.1038/ncomms12044 (PMC4932188; doi:10.1038/ncomms12044)
Supplement: Supplementary Information — Supplementary Figures 1-17 and Supplementary Tables 1-2 [file ncomms12044-s1.pdf]

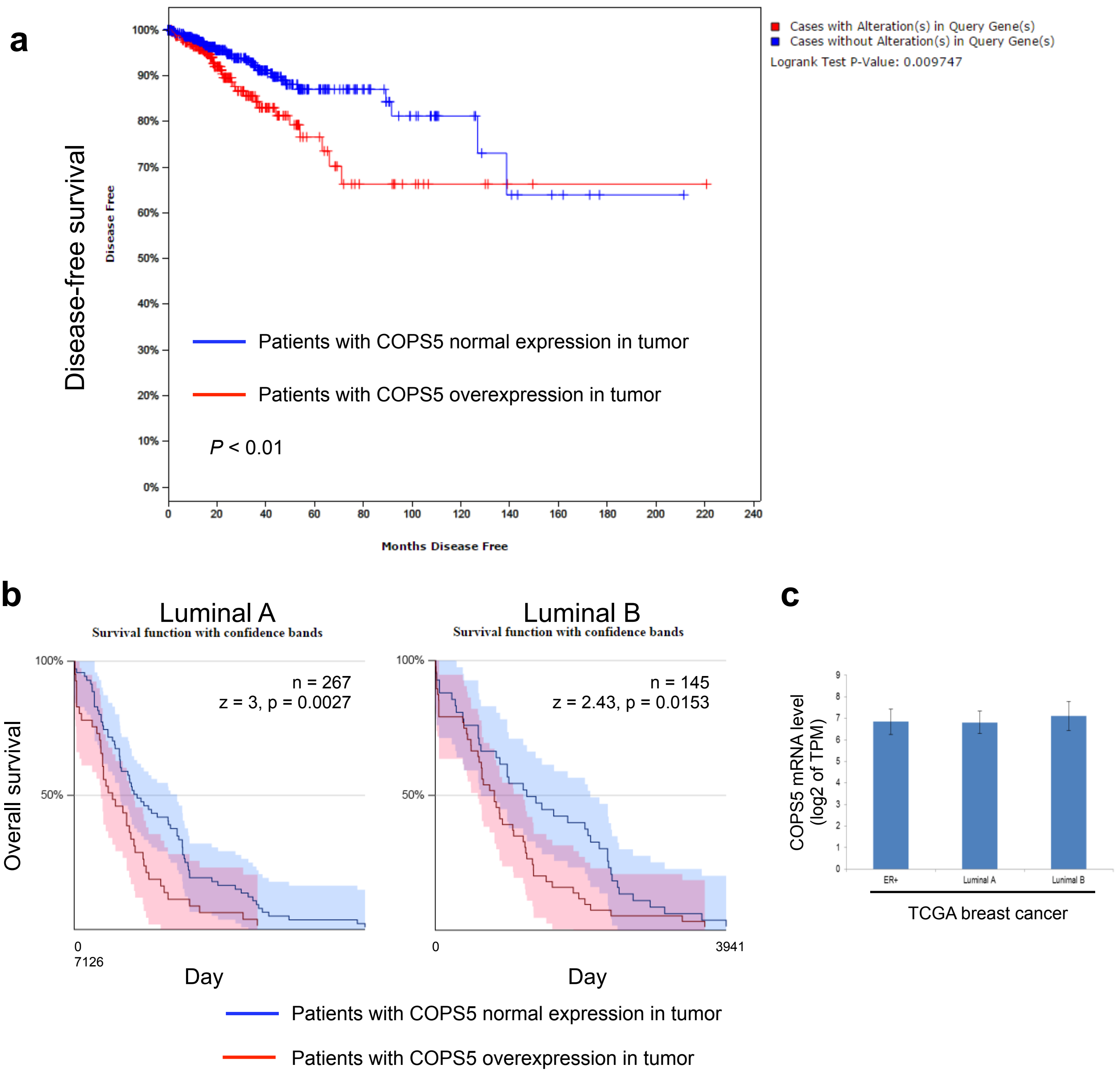

Supplementary Fig. 1. *COPS5*-overexpressed breast cancer patients have poor clinical outcomes in the TGCA cohort. (a) Data visualization of disease-free survival of all breast cancer patients was acquired from the cBio portal (http://www.cbioportal.org/). **(b)** Kaplan-Meier curves of overall survival of luminal A and B breast cancer patients based on the TCGA data portal (https://tcga-data.nci.nih.gov/tcga/tcgaHome2.jsp). **(c)** mRNA expression of COPS5 in ER+, luminal A and luminal B breast cancer patients.

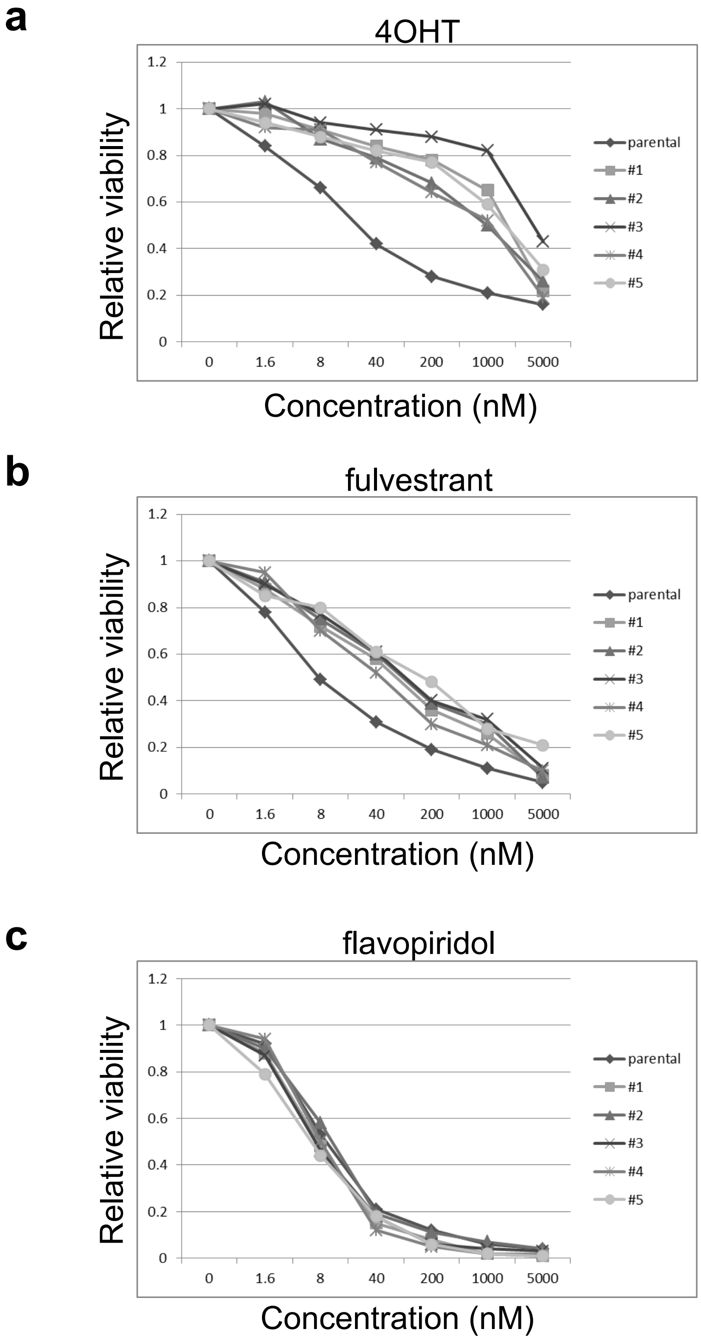

Supplementary Fig. 2. 4OHT-resistant MCF7 clones are selectively resistant to ERα antagonists. (a) IC50 curves of 4OHT-treatment (6 days). **(b)** IC50 curves of fulvestrant-treatment (6 days). **(c)** IC50 curves of flavopiridol-treatment (3 days).

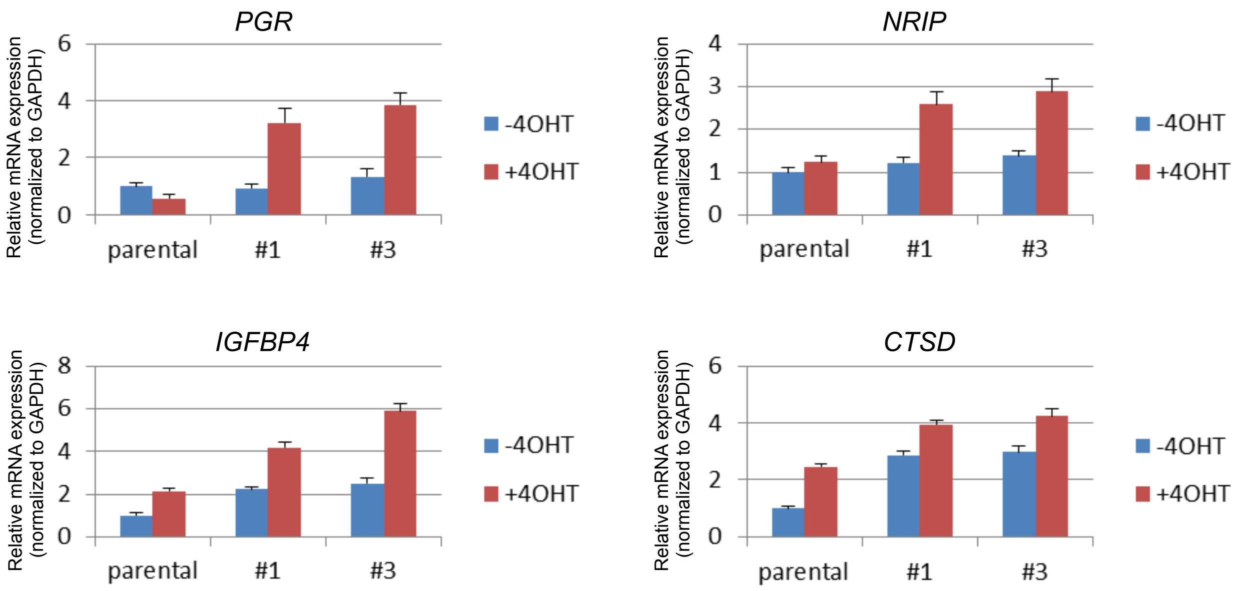

Supplementary Fig. 3. Quantitative RT-PCR analysis of ERα target genes in MCF7 parental and 4OHT-resistant clones. *PGR, NRIP, IGFBP4* and *CTSD* mRNA expression in MCF7 parental and 4OHT-resistant clones #1 and 3 was examined after treatment with 500nM 4OHT for 48 hours. Data are presented as mean±SEM of three technical replicates.

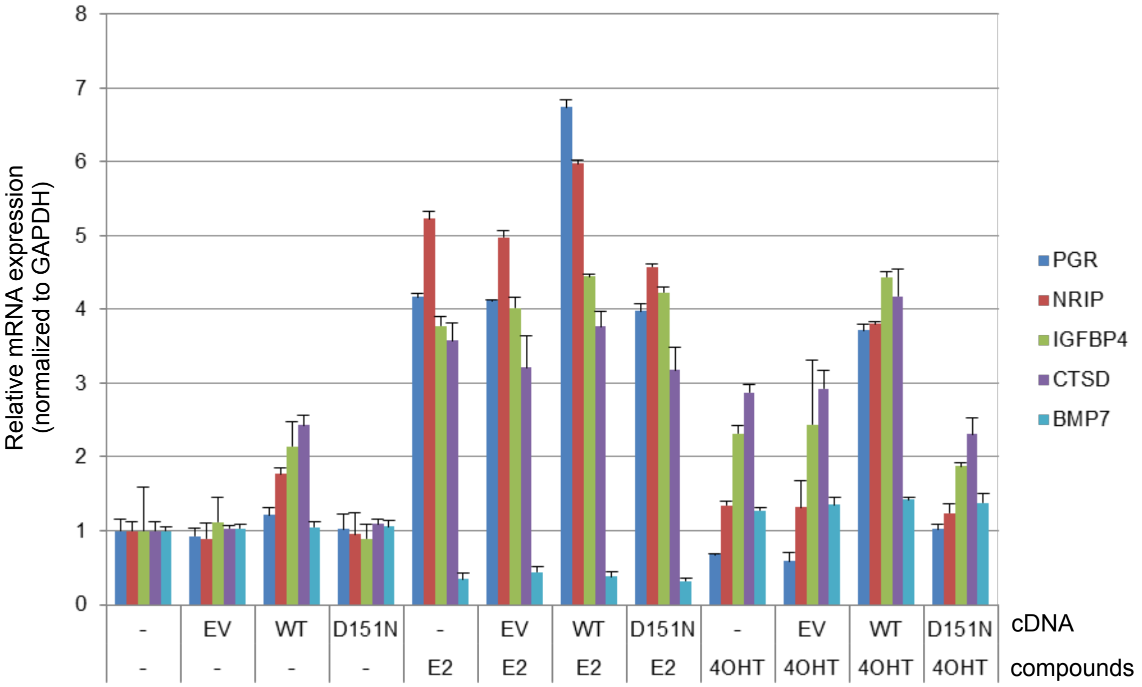

Supplementary Fig. 4. Quantitative RT-PCR analysis of ERα target genes in MCF7 cells. *PGR, NRIP, IGFBP4, CTSD* and *BMP7* mRNA expression was examined by ERα ligand treatment (48 hours) in MCF7 cell lines stably engineered with empty vector (EV), WT or D151N COPS5 cDNA. Data are presented as mean±SEM of three technical replicates. E2: 1 nM, 4OHT: 500 nM.

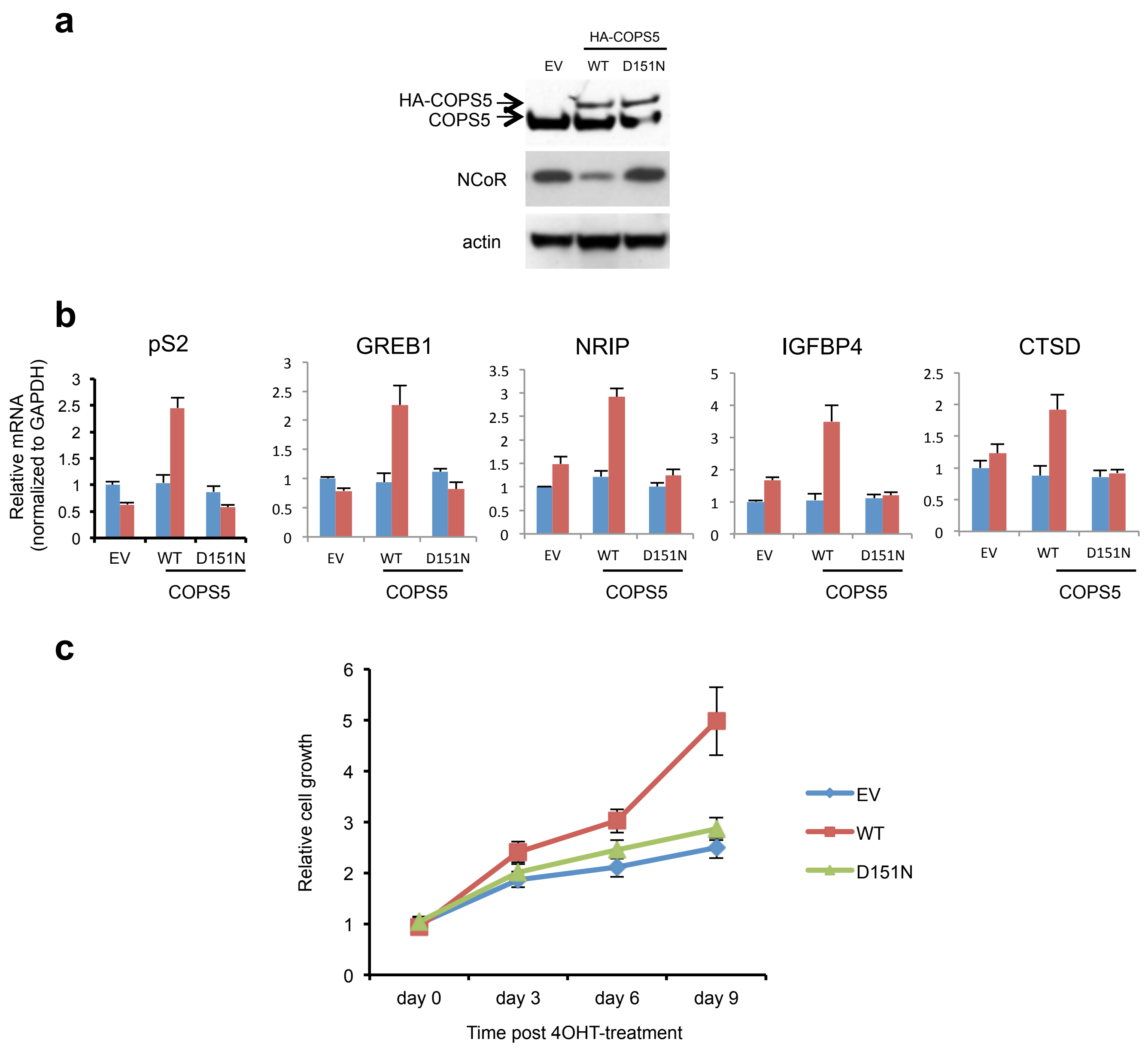

Supplementary Fig. 5. Overexpression of WT COPS5 confers resistance to 4OHT in T47D cells. (a) stable expression of WT but not D151N COPS5 leads to downregulation of NCoR in T47D cells. **(b)** ERα target genes *pS2, GREB1, NRIP, IGFBP4 and CTSD* are upregulated by 4OHT in WT-COPS5-overexpressing T47D cells. **(c)** 2 μM 4OHT-treatment does not completely inhibit the growth of COPS5-overexpressing T47D cells. mRNA of *pS2* was normalized to that of *GAPDH*. Data were shown as mean±SEM of three biological replicates. WT COPS5: T47D engineered with WT COPS5 cDNA; D151N COPS5: T47D engineered with D151N COPS5 cDNA.

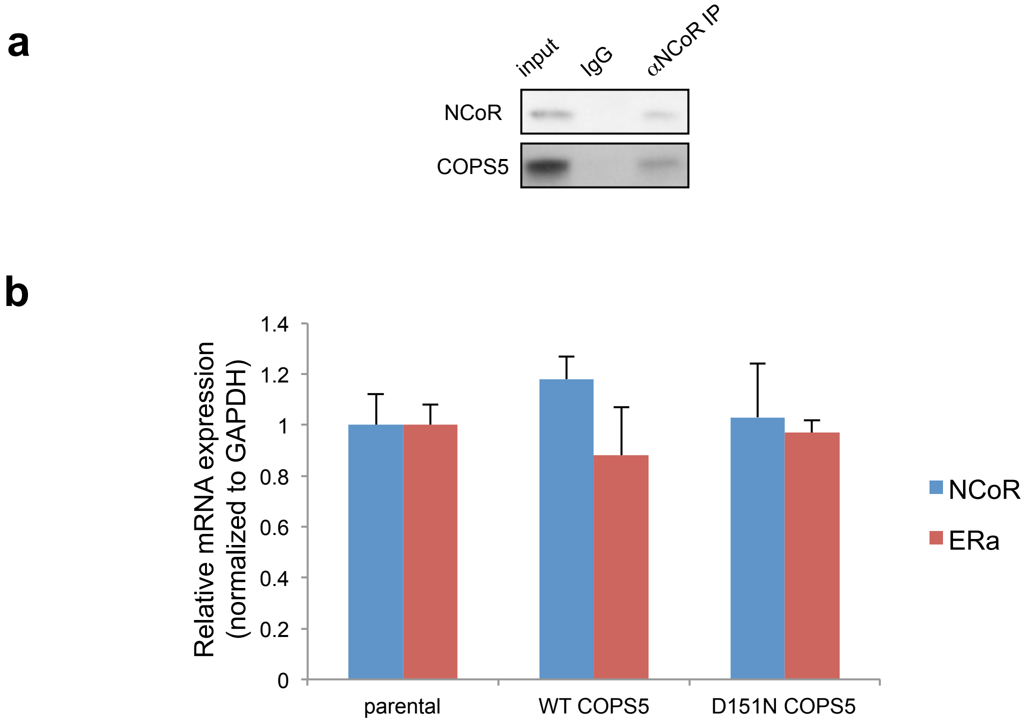

Supplementary Fig. 6. Interactions between NCoR and COPS5. (a) Western blot analysis of proteins that were co-immunoprecipitated with anti-NCoR antibodies in MCF7 parental cells. **(b)** mRNA expression of NCoR and ERa in COPS5-engineered MCF7 cells. mRNA of *COPS5* and *ERa* was normalized to that of *GAPDH* and relative to *COPS5* expression in the parental cell line which was set as 1. Data were shown as mean±SEM of three biological replicates. WT COPS5: MCF7 engineered with WT COPS5 cDNA; D151N COPS5: MCF7 engineered with D151N COPS5 cDNA.

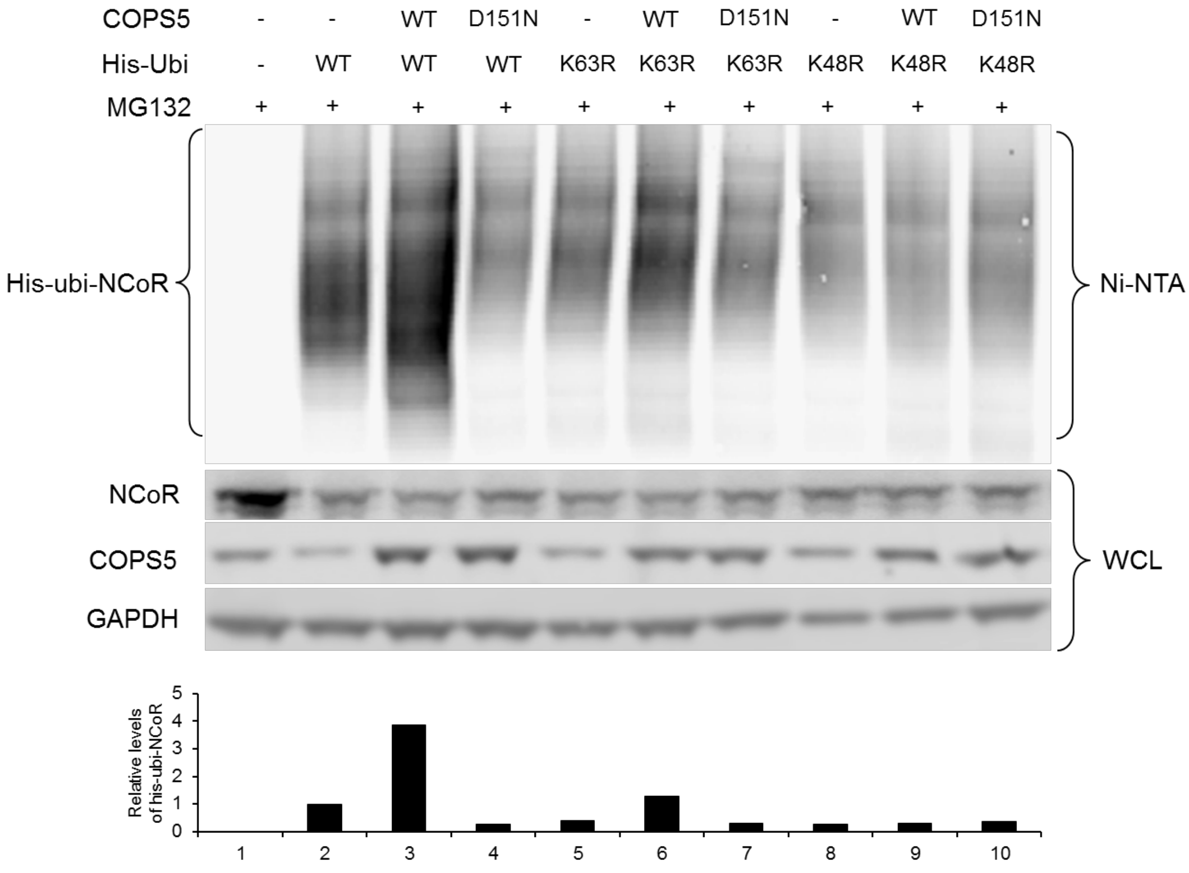

Supplementary Fig. 7. *In vivo* ubiquitination assay in HEK293T cells with overexpression of WT or D151N COPS5. WT, K48R and K63R His-ubi were used in the experiments. Proteins pulled down by Ni-NTA beads and existed in whole cell lysates (WCL) are tested by Western blot using antibodies shown on the left. His-Ubi: His_6-tagged ubiquitin, Ubi-NCoR, polyubiquitinated NCoR protein. NIH ImageJ was used for Western blot quantification.

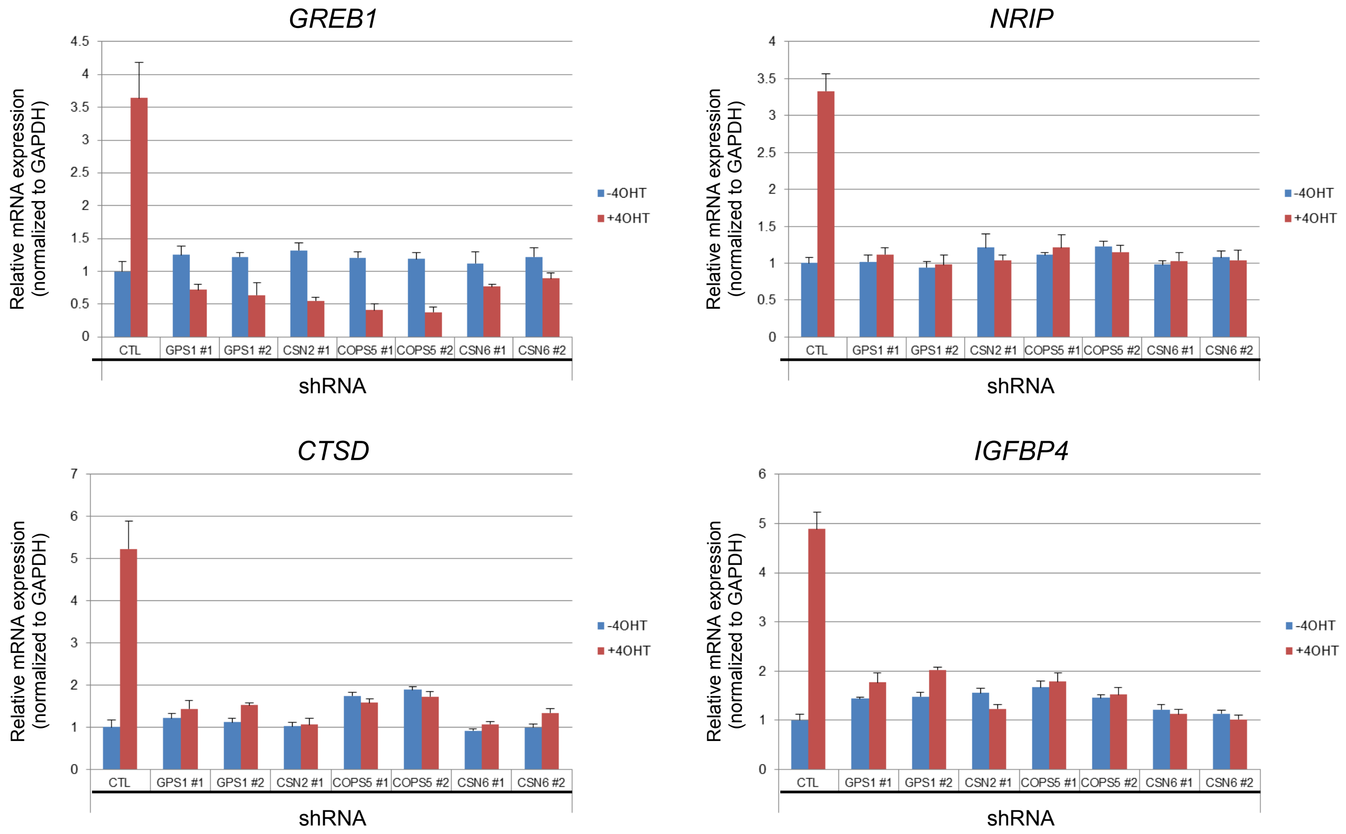

Supplementary Fig. 8. Quantitative RT-PCR analysis of ERα target genes in MCF7 cells. *GREB1, NRIP, IGFBP4* and *CTSD* mRNA expression was examined in the MCF7 cell lines shown in Fig. 4e in response to 4OHT (500 nM) for 48 hours. Data are presented as mean$\pm$SEM of three technical replicates. CTL: control.

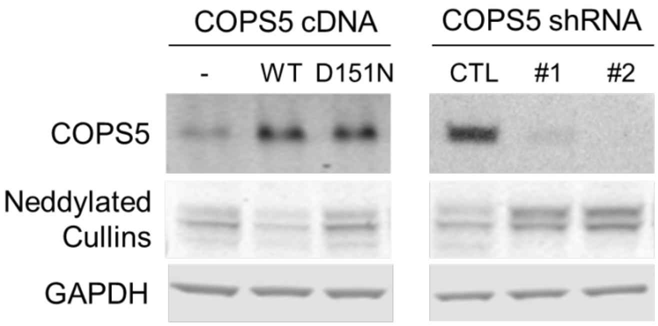

Supplementary Fig. 9. Western blot analysis of MCF7 cells upon overexpression of COPS5 cDNAs or COPS5 shRNAs. Left panels: paretnal MCF7 cells; right panels: 4OHT-resistant COPS5-overexpressed MCF7 clone #3. Anti-nedd8 antibody Y297 (ab81264, Abcam) was used to detect neddylated cullins (~95 kD). CTL: control.

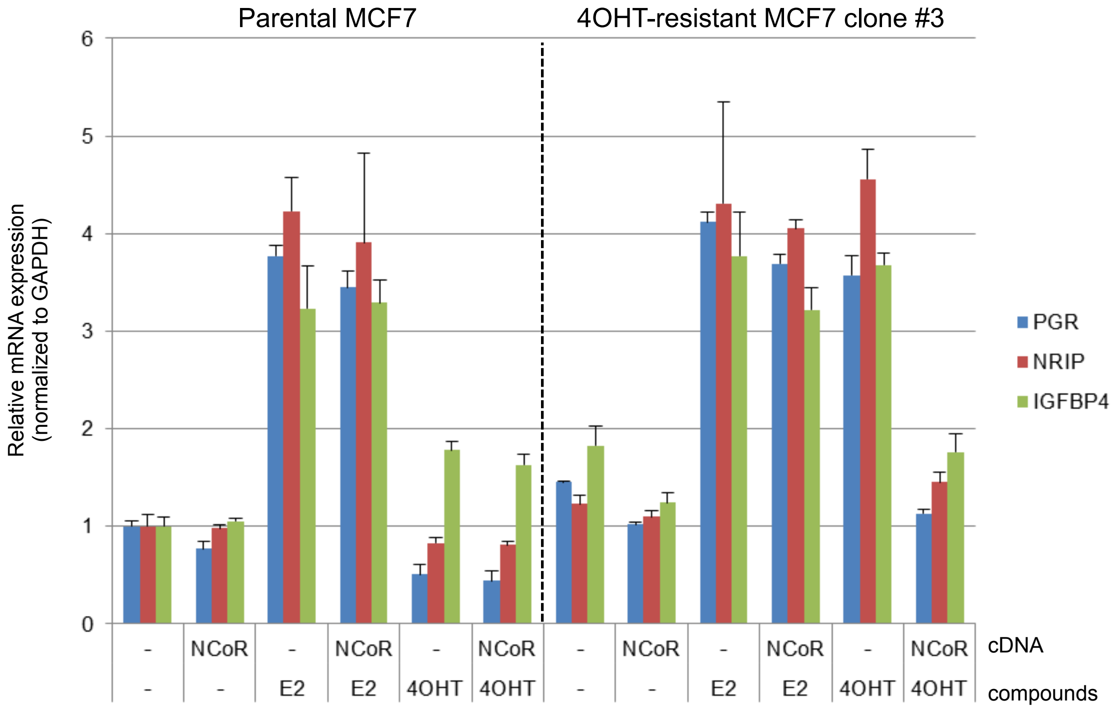

Supplementary Fig. 10. Quantitative RT-PCR analysis of ERα target genes in MCF7 cells. *PGR, NRIP* and *IGFBP4* mRNA expression was examined in parental and 4OHT-resistant MCF7 clones with expression of NCoR. Ligand treatment: E2: 1 nM, 4OHT: 500 nM, 48 hrs. Data are presented as mean±SEM of three technical replicates.

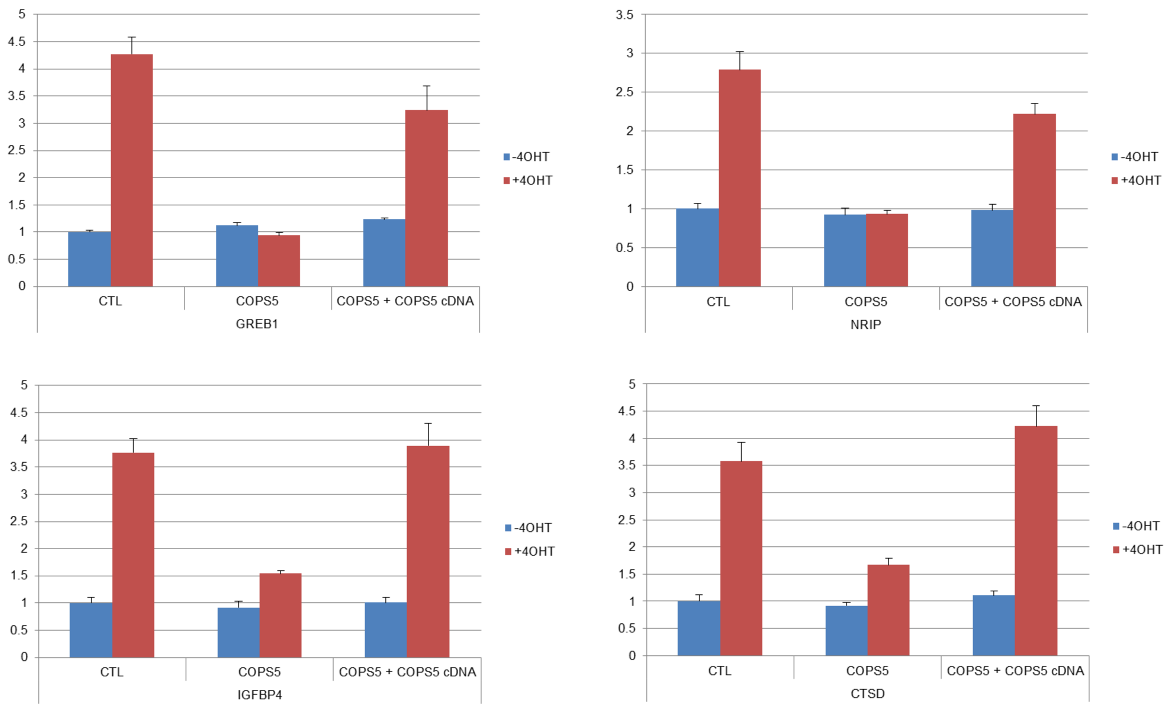

Supplementary Fig. 11. Quantitative RT-PCR analysis of ERα target genes in MCF7 cells. *GREB1, NRIP, IGFBP4* and *CTSD* mRNA expression was examined in the MCF7 cell lines shown in Fig. 5c in response to 4OHT (500 nM, 48 hours). Data are presented as mean±SEM of three technical replicates.

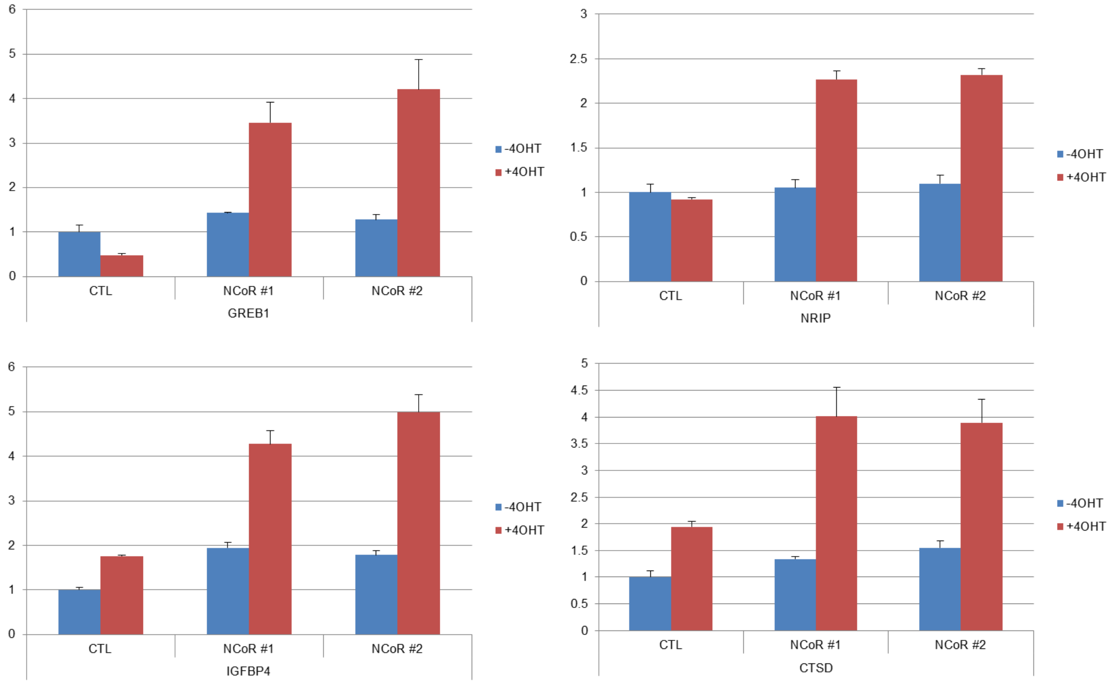

Supplementary Fig. 12. Quantitative RT-PCR analysis of ERα target genes in MCF7 cells. *GREB1, NRIP, IGFBP4* and *CTSD* mRNA expression was examined in the MCF7 cell lines shown in Fig. 5e in response to 4OHT (500 nM, 48 hours). Data are presented as mean±SEM of three technical replicates.

TCGA invasive breast carcinoma (provisional)

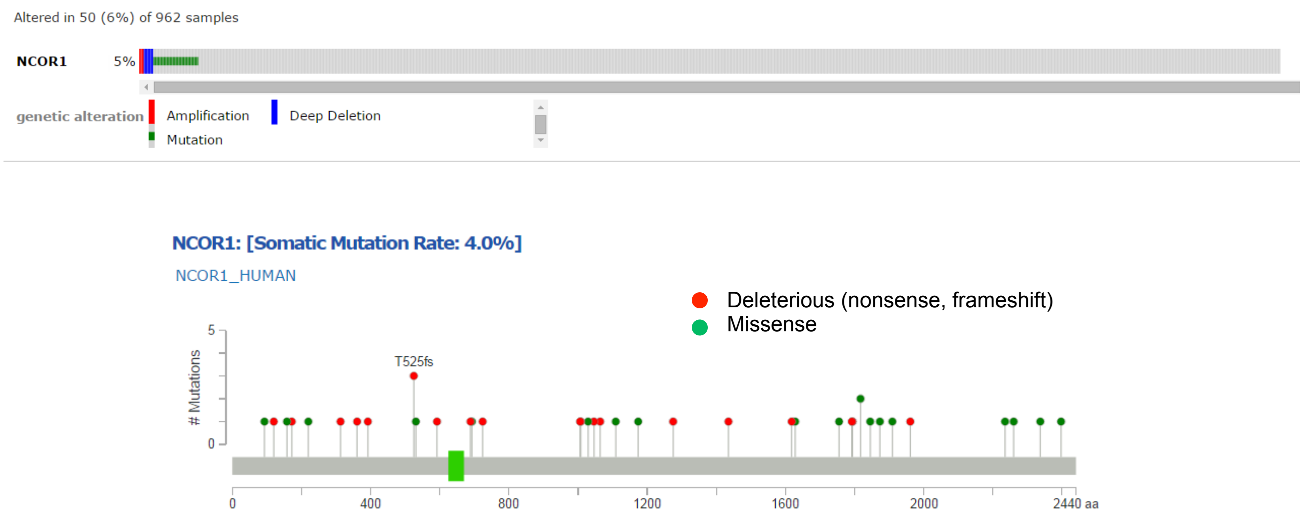

Supplementary Fig. 13. Genomic alterations of *NCOR1* in TCGA breast cancer. Data visualization was acquired from the cBio portal (http://www/cbioportal.org/).

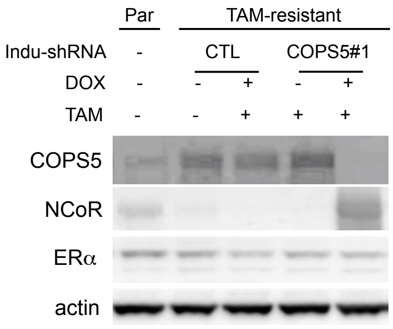

Supplementary Fig. 14. Western blot analysis of MCF7 tumors. Tumor samples isolated from the endpoint (day 42) of Fig. 7e were examined for protein expression of COPS5, NCoR, ERα and actin (loading control) by Western blot analysis. "*Par*+TAM-Dox" and "*TAM-resistant/shCTL*+TAM-Dox" tumors could not be used due to tumor size and quality issues. Par, parental; TAM: tamoxifen; Indu-shRNA: inducible shRNA; CTL: control; DOX: doxycycline.

a

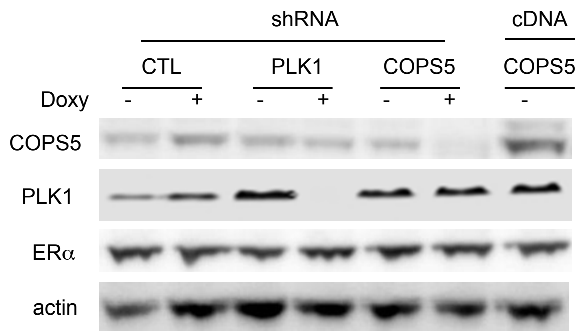

b

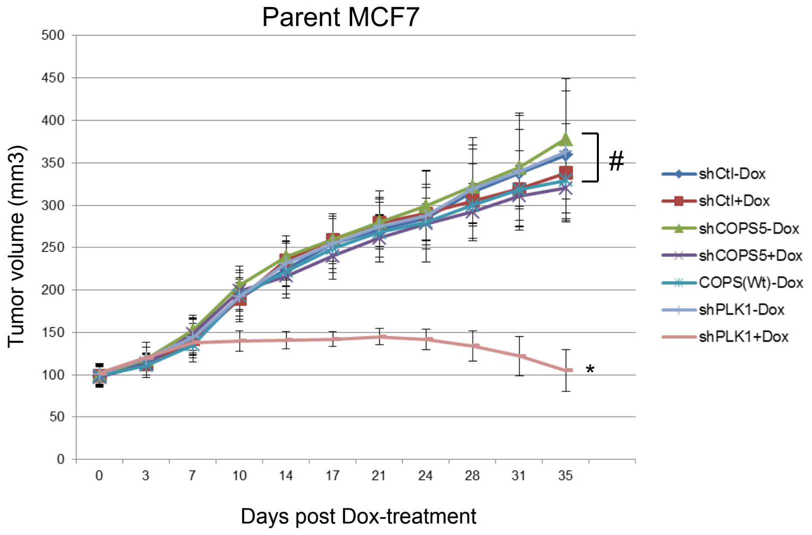

Supplementary Fig. 15. Xenograft models of parental MCF7 cell lines with genetic modulation of COPS5 expression **(a)** Western blot analysis of COPS5, PLK1, ERα and actin (loading control) in MCF7 tumor samples. Samples were collected with/without doxycycline treatment for 7 days**. (b)** Volumes of the MCF7 Xenograft tumors upon treatment as indicated. Data are presented as mean+SD of 5 mice each group growth in mice. t-test for samples at day 35: * P value <0.01 with others; # P value(s) >0.05 with others.

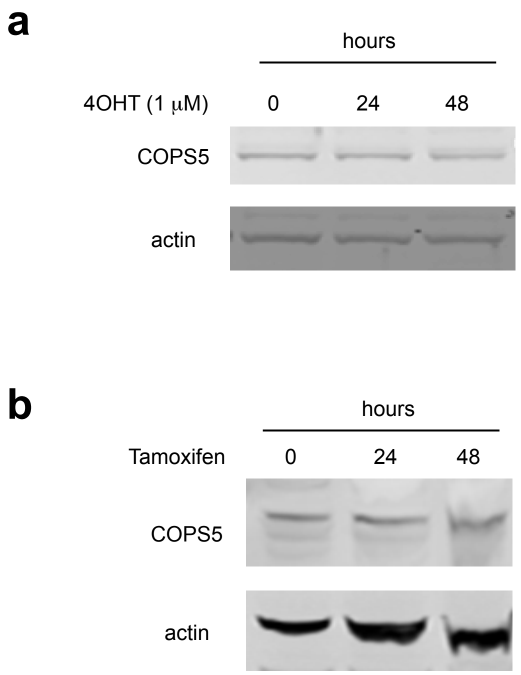

Supplementary Fig. 16. Acute treatment of MCF7 cells or tumors with 4OHT/ tamoxifen did not alter COPS5 expression. (a) MCF7 cells were treated with 1 μM 4OHT for 1 day and 2 days *in vitro*. Cell lysates were then subject to Western blot analysis for COPS5 and actin (loading control). **(b)** Mouse xenograft tumors of MCF7 were treated with single dose of tamoxifen (S.C., 40 mg/kg). Then tumor lysates were prepared 1 day and 2 days after dosing, and subject to Western blot analysis for COPS5 and actin (loading control).

Fig. 1i

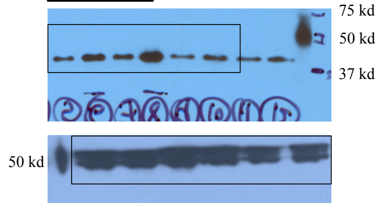

Fig. 2a

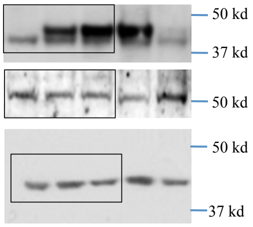

Fig. 3a

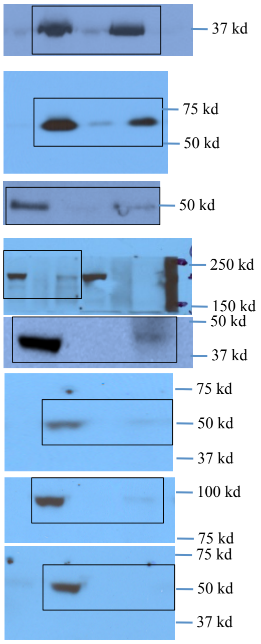

Fig. 3b

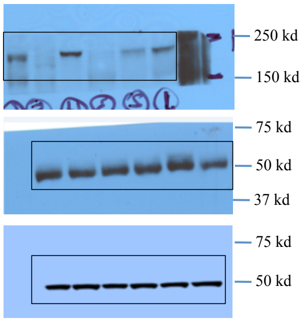

Fig. 4a

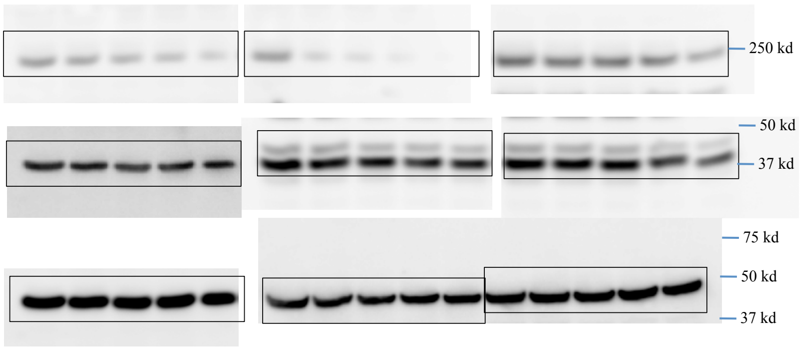

Fig. 4c

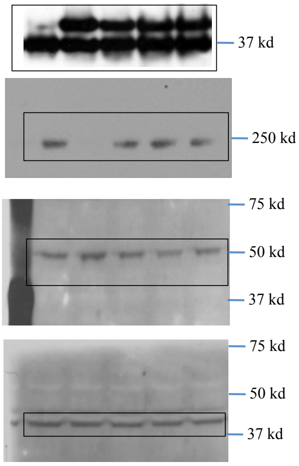

Fig. 4d

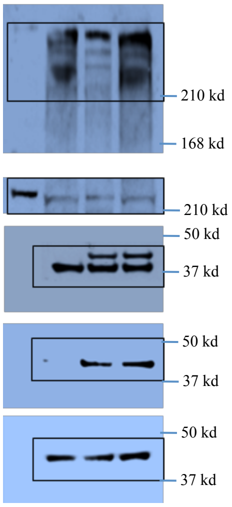

Fig. 4e

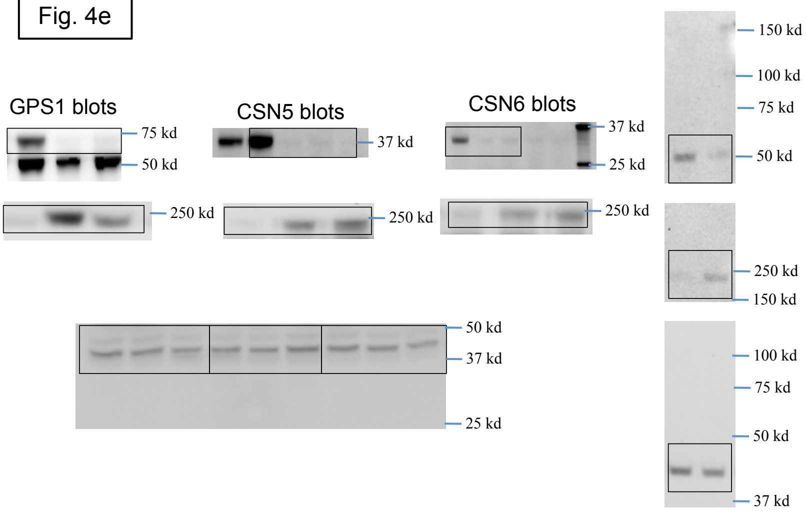

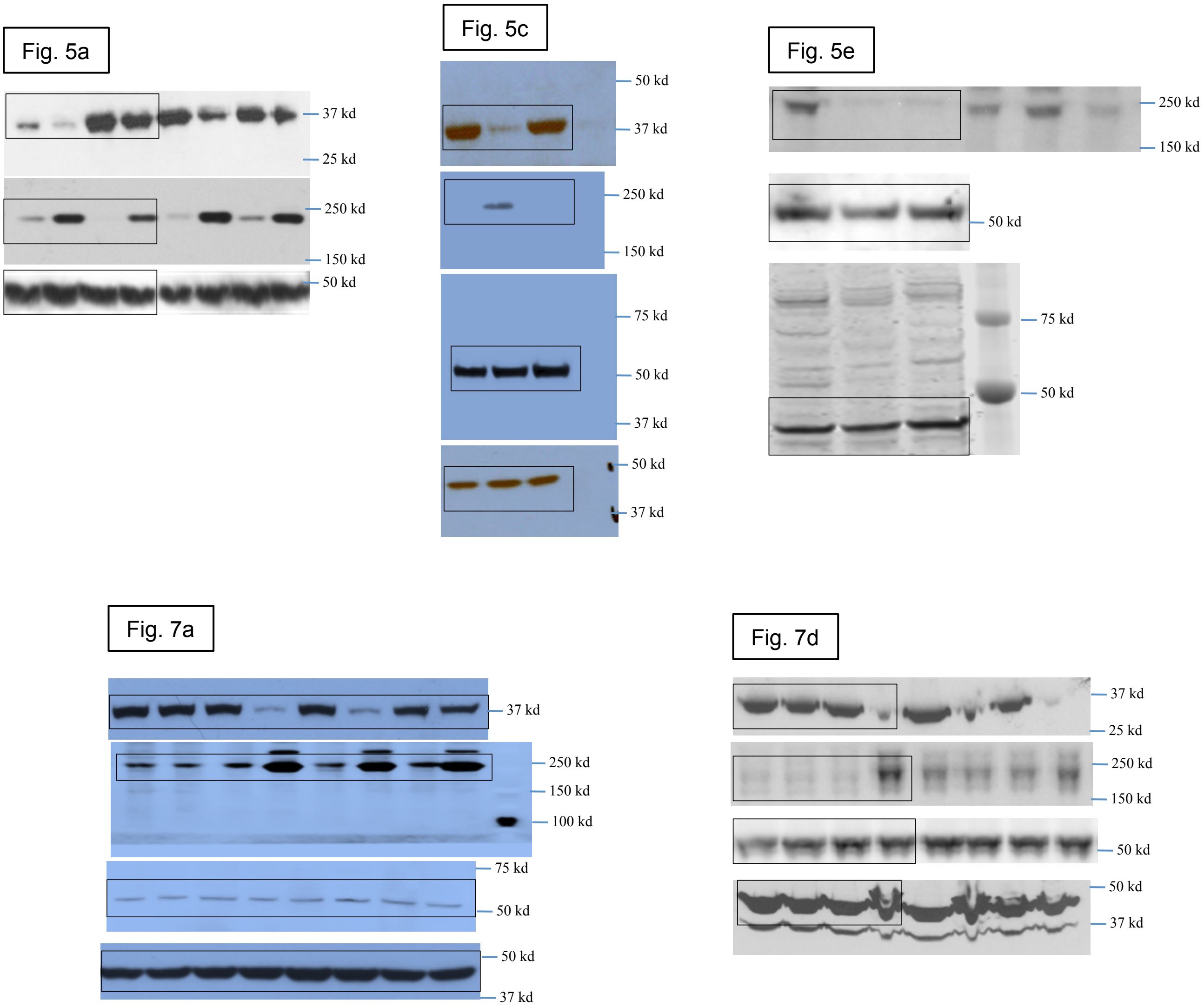

Supplementary Fig. 17. Uncropped western blot images presented in this manuscript. Labeling above each image indicates the corresponding figure in the main manuscript.

Supplementary Table 1. High amplification of *COPS5* (CN>12) is enriched in ERα-positive invasive breast carcinomas (TCGA)

	**ESR1 mRNA > 5 (ERα-positive)**	**ESR1 mRNA < 4 (ERα-negative)**	***Row Totals***
**COPS5 CN > 12**	27 (21.09) [1.66]	1 (6.91) [5.06]	28
**COPS5 CN < 12**	717 (722.91) [0.05]	243 (237.09) [0.15]	960
Column Totals	744	244	988 (Grand Total)

The table provides the following information: the observed totals, (the expected totals) and [the chi-square statistic for each cell]. The P value is 0.008548. This result is significant at $P < 0.05$.

Supplementary Table 2. IHC score of COPS5, NCoR and ERα in breast tumor samples

Groups	Patients No.	COPS5	NCoR	ERα
TAM-refractory (n=30)				
	Patient #1	+	-	+
	Patient #2	+	-	+
	Patient #3	+	-	+++
	Patient #4	+	-	+
	Patient #5	-	+	-
	Patient #6	++	+	+
	Patient #7	+	-	+
	Patient #8	+	-	+
	Patient #9	-	+	+
	Patient #10	+	-	+
	Patient #11	+	-	+
	Patient #12	+	-	+++
	Patient #13	++	-	+
	Patient #14	++	+	++
	Patient #15	+++	++	++
	Patient #16	+	-	+
	Patient #17	++	+	+
	Patient #18	+	++	++
	Patient #19	++	-	+
	Patient #20	+	++	+
	Patient #21	+++	-	++
	Patient #22	+	-	+++
	Patient #23	-	-	++
	Patient #24	+	+	+
	Patient #25	+	-	++
	Patient #26	++	-	+++
	Patient #27	+++	+	++
	Patient #28	++	-	+
	Patient #29	-	-	++
	Patient #30	+	+	+
TAM-untreated (n=31)				
	Patient #1	-	+	+
	Patient #2	-	+	+
	Patient #3	+	-	++
	Patient #4	-	+	+
	Patient #5	-	+	-
	Patient #6	-	-	+++
	Patient #7	-	-	+
	Patient #8	-	+	+
	Patient #9	-	+	+
	Patient #10	-	+	+++
	Patient #11	++	+	+
	Patient #12	+	-	+
	Patient #13	-	+	++
	Patient #14	-	+	++
	Patient #15	-	+	+
	Patient #16	-	+	+
	Patient #17	+	++	+
	Patient #18	+	++	+
	Patient #19	-	+	+
	Patient #20	-	+	++
	Patient #21	+	+	+
	Patient #22	-	+	+
	Patient #23	-	-	+
	Patient #24	-	-	+
	Patient #25	+	++	++
	Patient #26	-	+	+
	Patient #27	-	+	++
	Patient #28	++	+	+
	Patient #29	-	-	+++
	Patient #30	-	+	-
	Patient #31	-	-	++

IHC score is described in Methods.
+++: very strong positive
++: strong positive
+: positive
-: negative
